# The ZIP Code of Vesicle Trafficking in Apicomplexa: SEC1/Munc18 and SNARE Proteins

**DOI:** 10.1128/mBio.02092-20

**Published:** 2020-10-20

**Authors:** Hugo Bisio, Rouaa Ben Chaabene, Ricarda Sabitzki, Bohumil Maco, Jean Baptiste Marq, Tim-Wolf Gilberger, Tobias Spielmann, Dominique Soldati-Favre

**Affiliations:** a Department of Microbiology and Molecular Medicine, CMU, Faculty of Medicine, University of Geneva, Geneva, Switzerland; b Department of Molecular Biology and Immunology, Bernhard Nocht Institute for Tropical Medicine, Hamburg, Germany; c Centre for Structural Systems Biology, Hamburg, Germany; d Biology Department, University of Hamburg, Hamburg, Germany; Albert Einstein College of Medicine

**Keywords:** Apicomplexa, *Toxoplasma gondii*, *Plasmodium falciparum*, microneme, exocytosis, apicoplast, membrane fusion, pellicle, inner membrane complex, SNARE, small GTPases, syntaxin, Vps45, SEC1/Munc18, apicomplexan parasites, rhoptry

## Abstract

Apicomplexans are obligate intracellular parasites harboring three sets of unique secretory organelles termed micronemes, rhoptries, and dense granules that are dedicated to the establishment of infection in the host cell. Apicomplexans rely on the endolysosomal system to generate the secretory organelles and to ingest and digest host cell proteins. These parasites also possess a metabolically relevant secondary endosymbiotic organelle, the apicoplast, which relies on vesicular trafficking for correct incorporation of nuclear-encoded proteins into the organelle. Here, we demonstrate that the trafficking and destination of vesicles to the unique and specialized parasite compartments depend on SNARE proteins that interact with tethering factors. Specifically, all secreted proteins depend on the function of SLY1 at the Golgi. In addition to a critical role in trafficking of endocytosed host proteins, TgVps45 is implicated in the biogenesis of the inner membrane complex (alveoli) in both Toxoplasma gondii and Plasmodium falciparum, likely acting in a coordinated manner with Stx16 and Stx6. Finally, Stx12 localizes to the endosomal-like compartment and is involved in the trafficking of proteins to the apical secretory organelles rhoptries and micronemes as well as to the apicoplast.

## Abstract (precis)

The phylum of Apicomplexa groups medically relevant parasites such as those responsible for malaria and toxoplasmosis. As members of the Alveolata superphylum, these protozoans possess specialized organelles in addition to those found in all members of the eukaryotic kingdom. Vesicular trafficking is the major route of communication between membranous organelles. Neither the molecular mechanism that allows communication between organelles nor the vesicular fusion events that underlie it are completely understood in Apicomplexa. Here, we assessed the function of SEC1/Munc18 and SNARE proteins to identify factors involved in the trafficking of vesicles between these various organelles. We show that SEC1/Munc18 in interaction with SNARE proteins allows targeting of vesicles to the inner membrane complex, prerhoptries, micronemes, apicoplast, and vacuolar compartment from the endoplasmic reticulum, Golgi apparatus, or endosomal-like compartment. These data provide an exciting look at the “ZIP code” of vesicular trafficking in apicomplexans, essential for precise organelle biogenesis, homeostasis, and inheritance.

## INTRODUCTION

In eukaryotic cells, interorganelle communication is accomplished mostly by trafficking of vesicles that bud and get transported along the cytoskeleton to dock and fuse with target membranes (1). Vesicular membrane fusion is a universal process in eukaryotic cells, and the correct trafficking of vesicles is ensured by unique identifiers. These ZIP codes are composed of three types of molecules: small GTPases, phosphatidylinositol phospholipids, and soluble N-ethylmaleimide-sensitive factor attachment protein receptors (SNAREs) (2, 3). Sec1/Munc18-like proteins tightly regulate SNARE complex assembly by particularly binding to syntaxin (Stx)-like SNARE proteins (4).

Toxoplasma gondii and Plasmodium falciparum belong to the phylum of Apicomplexa that groups numerous parasites which are responsible for severe diseases in humans and animals. These parasites are highly polarized cells that harbor an apical complex composed of specialized secretory organelles called micronemes and rhoptries (5) that participate in motility, invasion and egress from infected cells, as well as modulation of host cellular responses (6–8).

In T. gondii, the endomembrane system has been actively investigated (9, 10), and the endosomal-like compartment (ELC) is a central hub in vesicular trafficking, which accepts and dispatches vesicles from the *trans*-Golgi network (TGN) to plasma membrane and a lysosome-like organelle termed the vacuolar compartment (VAC) (11). ELC is the sorting platform for the proteins trafficking to the micronemes and the rhoptries (12) and also the compartment where rhoptry and microneme proteins undergo aspartyl protease 3 (ASP3)-dependent maturation (13). These results suggest that T. gondii repurposes parts of the machinery typically involved in endocytosis for the biogenesis of these secretory organelles (9). The machinery involved in endocytosis in apicomplexans is not well understood, yet it is unquestionably critical during intraerythrocytic-stage development of the malaria parasites (14–16). In this context, endocytosis is required for the acquisition of host cell cytosolic proteins, which are internalized across the parasitophorous vacuole membrane (PVM) and the parasite plasma membrane, presumably at the level of the micropore (17, 18) or the cytostome (18, 19), to be finally dispatched in the lysosomal-like compartment for degradation (15, 20, 21). This compartment refers to the food vacuole in P. falciparum (22, 23) and to the VAC in T. gondii (24, 25). The molecular mechanisms involved in protein trafficking from the cytosol of the host to the VAC are largely unknown (18). Taken together, the ELC is the compartment where endocytic and exocytic systems intersect (21). Sorting of proteins at the ELC is temporally orchestrated and depends on several tethering molecules, including DrpB (26), HOPS, CORVET (27), clathrin (28), and the small GTPases referred to as Rab5A and Rab5C in T. gondii (11, 29).

Remarkably, unlike most cells, the apicomplexan parasites do not replicate by binary fission, but instead, produce multiple progeny within the mother cell prior to cytokinesis. This process is referred to as schizogony when daughter cell nuclei are formed before membrane assembly or endopolygeny when nuclei and membranes develop in parallel. T. gondii tachyzoites divide by endodyogeny, meaning that only two daughters are produced per each mother (30–33), although they remain connected via a tubular network and residual body at the posterior pole of the parasite (34). Essential for parasite morphology and movement (30, 32), the membrane-cytoskeletal scaffold known as the inner membrane complex (IMC) is composed of flattened vesicles presumably derived from the *trans*-Golgi. The IMC forms the parasite pellicle together with the plasma membrane. Another feature of most apicomplexans is the presence of a plastid-like organelle surrounded by four membranes termed the apicoplast (35). This organelle originates from a secondary endosymbiotic event and hosts several metabolic pathways, including fatty acids, isoprenoids, and heme biosynthesis (36). The majority of apicoplast resident proteins are encoded in the nuclear genome and are targeted through the endoplasmic reticulum (ER) to the apicoplast via a bipartite N-terminal targeting signal (37). In contrast, nuclear-encoded transmembrane proteins resident in the apicoplast traffic through the Golgi apparatus (38).

In model organisms, Sec1/Munc18 and SNARE proteins are needed for all vesicle fusion events and are critical for vesicular trafficking in the cell (3). In this study, we have functionally characterized Sec1/Munc18 and SNARE proteins and compared the role of the Sec1/Munc18 protein vacuolar sorting protein 45 (Vps45) in the phylogenetically related parasites T. gondii and P. falciparum. We uncovered that the role of Vps45 in endocytosis, previously reported in malaria parasites (15), is conserved in T. gondii, allowing VAC mediated digestion of host ingested proteins. In addition, TgVps45 and PfVps45 share a critical role in IMC biogenesis, indicating (a developmental stage-dependent) dual role of this protein. Moreover, we demonstrate that the ELC function critically depends on several SNARE proteins. Overall, we dissect here some components of the ZIP code for vesicular trafficking to key compartments of the parasite linked to the specialized secretory organelles, the IMC, and the apicoplast.

## RESULTS

### SLY1 is needed to regulate the function of the Golgi.

**FIG 1:**
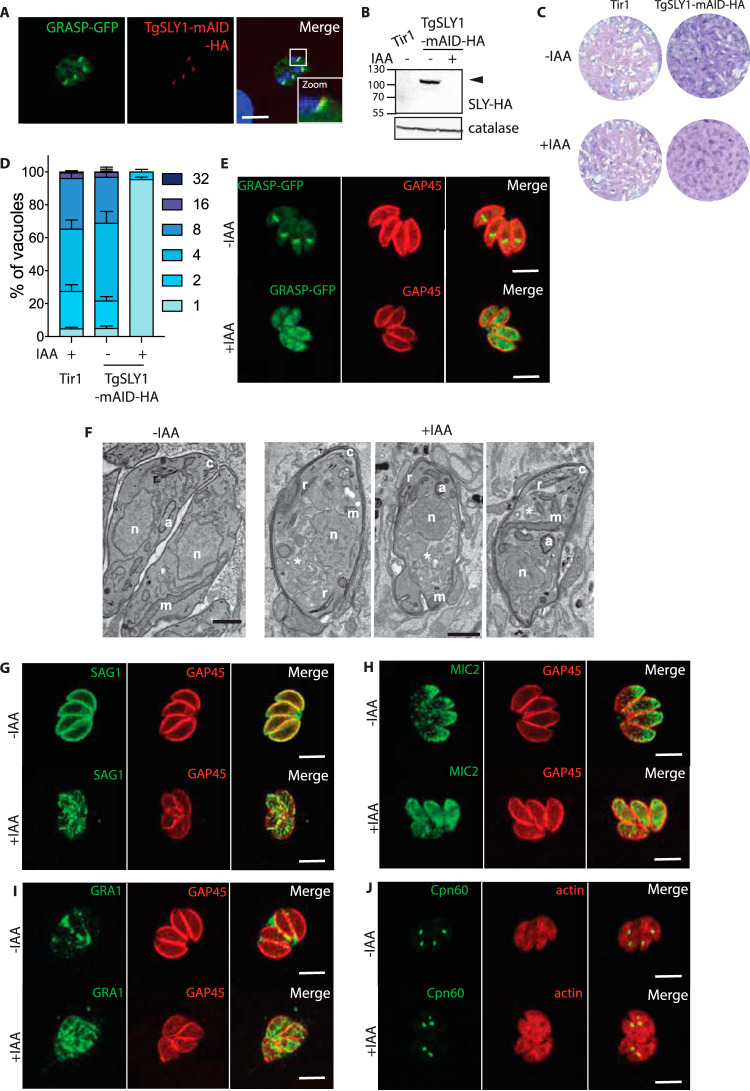
Toxoplasma gondii SLY mediates ER to Golgi vesicle transport. (A) Indirect immunofluorescence assay (IFA) showing the endogenously C-terminally tagged SLY localized to the Golgi. GRASP-GFP, parasite Golgi marker. (B) Western blot showing auxin (IAA)-induced SLY-mAID-HA degradation in intracellular parasites within 2 hours. Catalase, loading control. (C) Plaque assay of the TgSLY-mAID-HA and Tir1 parental strain in the presence or absence of IAA. TgSLY-mAID-HA displays a severe defect in the lytic cycle in the presence of IAA. (D) Parasites lacking TgSLY are impaired in intracellular replication. Error bars represent the ± standard deviation (SD) from three independent experiments. (E) IFA of TgSLY-mAID-HA parasites ± IAA (24 hours). GRASP-GFP failed to localize to the Golgi upon TgSLY degradation. GAP45, marker of the parasite pellicle. Parasites were allowed to grow for 24 hours in the absence of IAA and incubated with IAA for another 24 hours. (F) Electron micrographs reveal severe morphological defects of the Golgi apparatus and endoplasmic reticulum in SLY1-iKD parasites (marked with asterisk) upon auxin treatment (+IAA) compared to the typical Golgi and ER marked with arrowheads in the control (–IAA). Some organelles are highlighted as a, apicoplast; c, conoid; n, nucleus; m, mitochondrion; and r, rhoptry. Scale bars = 1 μm. (G) IFA of TgSLY-mAID-HA parasites ± IAA (24 hours). Trafficking of the GPI-anchored SAG1 protein to the parasite plasma membrane is affected in the absence of TgSLY. GAP45, marker of the parasite pellicle. Parasites were allowed to grow for 24 hours in the absence of IAA and incubated with IAA for another 24 hours. (H) IFA of TgSLY-mAID-HA parasites ± IAA (24 hours). Trafficking of the microneme localized MIC2 protein is affected in the absence of SLY. GAP45, marker of the parasite pellicle. Parasites were allowed to grow for 24 hours in the absence of IAA and incubated with IAA for another 24 hours. (I) IFA of TgSLY-mAID-HA parasites ± IAA (24 hours). Trafficking of GRA1 protein to the dense granules is affected in the absence of TgSLY. GAP45, marker of the parasite pellicle. Parasites were allowed to grow for 24 hours in the absence of IAA and incubated with IAA for another 24 hours. (J) IFA of TgSLY-mAID-HA parasites ± IAA (24 hours). Trafficking of Cpn60 protein to the apicoplast is not affected in the absence of TgSLY. Actin, parasite cytosol. Parasites were allowed to grow for 24 hours in the absence of IAA and incubated with IAA for another 24 hours. Scale bars for all IFA = 7 μm.

The apicomplexans encode 4 Sec1/Munc18 proteins, including a putative orthologue of the suppressor of loss of YPT1 function (SLY1) (TGME49_213072, TgSLY) (Table 1). In yeast, SLY1 is involved in the trafficking of vesicles from the ER to the Golgi apparatus (39, 40). To localize and functionally characterize T. gondii SLY1 (TgSLY1), Ku80 knockout strain RH expressing the Tir1 protein was modified at the endogenous locus to fuse TgSLY1 with mAID-3xHA at the carboxyl terminus (TgSLY1-mAID-HA) (Fig. S1A). In transgenic parasites, TgSLY1-mAID-HA colocalizes with the Golgi marker, Golgi reassembly stacking protein (GRASP)-green fluorescent protein (GFP) (Fig. 1A and Fig. 1SB), migrates at the predicted size of ∼120 kDa, and is rapidly degraded upon addition of 3-indole acetic acid (IAA) (Fig. 1B). Plaque assays revealed a dramatic loss of fitness in parasites depleted in TgSLY1 (Fig. 1C) that is associated with a severe impact on parasite intracellular growth (Fig. 1D). In the absence of TgSLY1, parasites do not accomplish replication and are arrested as single parasites inside the vacuole postinvasion. Importantly, this replication defect is accompanied by a complete block in karyokynesis (Fig. S1C) but only partially resulting from impairment of centrosome duplication, which indicates that the phenotype cannot be only explained by a failure of the cell cycle checkpoint (Fig. S1D). Upon SLY depletion, GRASP-GFP expressed by transient transfection showed a dispersed localization in the parasite rather than the defined foci typical for the Golgi (Fig. 1E and Fig. S1C), indicating that SLY1-depleted parasites possess a disturbed Golgi apparatus or a complete absence of the organelle. Morphological analysis of the SLY1-depleted parasites by electron microscopy also demonstrated an aberrant appearance of the organelles involved in the secretory pathway and the absence of a distinguishable Golgi apparatus (Fig. 1F). Concomitantly, trafficking of the GPI-anchored protein SAG1 to the parasite plasma membrane, the transmembrane protein MIC2 to the micronemes, and the GRA1 to the dense granules, all known to depend on Golgi trafficking (41), is disrupted in the absence of SLY1 (Fig. 1G to I and Fig. S1E to G). Moreover, proteins such as GRA1 and MIC2, which belong to different secretory organelles, partially overlap in their signal in the absence of SLY1, demonstrating aberrant trafficking (Fig. S1H). In contrast, CPN60, an apicoplast-resident protein, which traffics via the ER but not through the Golgi (42), is unaffected (Fig. 1J and Fig. S1I).

**TABLE 1:** Core SNARE trafficking components are conserved between Apicomplexan parasites^a^^,^^b^

	ToxoDB	LOPIT	FS (Tg)	PlasmoDB	E (Pb)	Coc	Hae	Piro	Cryp
R									
	TGME49_270070	Golgi	−5.01	PBANKA_1218400	E	P	P	P	P
	TGME49_215420	NC	−4.66	PBANKA_1339900	E	P	P	P	P
	TGME49_299180	NC	−1.62	PBANKA_0811700	E	P	P	P	P
	TGME49_230430	PM-i	−0.76	PBANKA_1401700	D	P	P	P	P
	TGME49_248100	Golgi	−0.55	—	—	P	A	A	A
	TGME49_246610	—	−4.56	—	—	P	A	A	A
	TGME49_257520	PM-i	−1.06	—	—	P	A	P	P
Qa									
**Stx5**	TGME49_226600	Golgi	−4.5	PBANKA_1346800	N.A.	P	P	P	P
**Stx16**	TGME49_247930	Golgi	−3.2	PBANKA_1456400	E	P	P	P	P
	TGME49_209820	PM-i	N.A.	PBANKA_0307600	N.A.	P	P	P	P
**Stx12**	TGME49_204060	Golgi	−0.97	PBANKA_0608500	N.A.	P	P	P	P
	TGME49_220190	Golgi	−3.28	—	—	P	A	A	A
Qb									
	TGME49_223620	Golgi	−4.44	PBANKA_0936300	N.A.	P	P	A	P
	TGME49_251710	—	−0.39	—	—	P	A	A	A
	TGME49_217780	ER	−5.36	PBANKA_1415300	E	P	P	P	P
	TGME49_306640	PM-i	−4.11	PBANKA_1312300	E	P	P	P	P
	TGME49_242080	Golgi	−0.98	—	—	P	A	A	P
	TGME49_278160	—	−1.09	PBANKA_1450600	E	P	P	A	A
	TGME49_311320	—	−3.41	—	—	P	A	A	A
Qc									
	TGME49_205030	Golgi	−3.94	PBANKA_1209600	N.A.	P	P	A	P
**Stx6**	TGME49_300240	Golgi	−4.38	PBANKA_1316200	E	P	P	P	P
	TGME49_208010	ER	−3.84	—	—	P	A	A	A
	TGME49_253360	PM-i	−2.68	PBANKA_1035000	N.A.	P	P	P	P
	TGME49_300290	Golgi	−1.71	PBANKA_1244500	E	P	P	A	P
Qbc									
	TGME49_319940	Golgi	−4.44	PBANKA_1418800	N.A.	P	P	A	P

a SNARE proteins encoded in the genome of T. gondii were mined in the genome of other apicomplexan subgroups. LOPIT localization predictions in T. gondii were obtained from ToxoDB. The fitness score prediction in T. gondii was obtained from reference 88, while essentiality prediction in P. berghei was obtained from reference 89.

b P indicates the presence of the gene, and A indicates it absence. E indicates essential, D indicates dispensable, and N.A. no data available. Proteins investigated in this study are highlighted in bold.

10.1128/mBio.02092-20.1 FIG S1 (A) PCR demonstrates correct integration of SLY-mAID-HA. 9079/9080, 1,100 bp (wild-type locus). 9070/7081, 850 bp. (B) Indirect immunofluorescence assay (IFA) showing the endogenously C-terminally tagged SLY localization. Actin, parasite cytosol. (C) IFA of SLY-mAID-HA parasites + IAA (24 hours). GRASP-GFP failed to localize to the Golgi upon SLY degradation. GAP45, marker of the parasite pellicle. Parasites were incubated with IAA immediately after invasion (30 minutes to 1 hour). (D) IFA of SLY-mAID-HA parasites ± IAA (24 hours). Centrin1 showed duplication of centrosome in some parasites upon SLY degradation. GAP45, marker of the parasite pellicle. Parasites were incubated with IAA immediately after invasion (30 minutes to 1 hour). (E) IFA of SLY-mAID-HA parasites + IAA (24 hours). Trafficking of the GPI-anchored SAG1 protein to the parasite plasma membrane is affected in the absence of SLY. Parasites were incubated with IAA immediately after invasion (30 minutes to 1 hour). (F) IFA of SLY-mAID-HA parasites + IAA (24 hours). Trafficking of the microneme localized MIC2 protein is affected in the absence of SLY. Parasites were incubated with IAA immediately after invasion (30 minutes to 1 hour). (G) IFA of SLY-mAID-HA parasites + IAA (24 hours). Trafficking of GRA1 protein to the dense granules is affected in the absence of SLY. (H) IFA of SLY-mAID-HA parasites ± IAA (24 hours). MIC2 and GRA1 colocalize in parasites upon SLY degradation. MIC2, marker of the micronemes; GRA1, marker of the dense granules. Parasites were incubated with IAA immediately after invasion (30 minutes to 1 hour). (I) IFA of SLY-mAID-HA parasites + IAA (24 hours). Trafficking of the Cpn60 protein to the apicoplast is not affected in the absence of SLY. Parasites were incubated with IAA immediately after invasion (30 minutes to 1 hour). Download FIG S1, PDF file, 0.4 MB. Copyright © 2020 Bisio et al. 2020 Bisio et al. This content is distributed under the terms of the Creative Commons Attribution 4.0 International license.

### Vps45 is implicated in IMC formation in both Toxoplasma gondii and Plasmodium falciparum.

**FIG 2:**
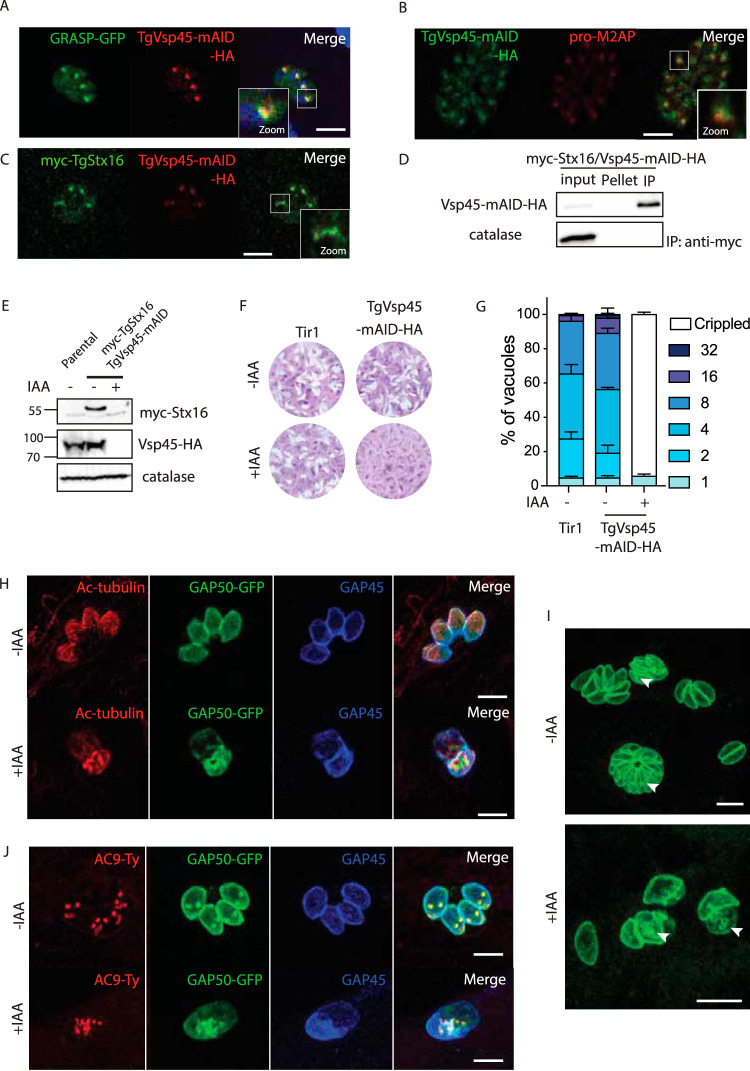
Vsp45 is implicated in the IMC formation *of* Toxoplasma gondii (A) C-terminal epitope tagging of TgVsp45 at the endogenous locus partially colocalizes with the Golgi marker GRASP-GFP. (B) C-terminal epitope tagging of Vsp45 at the endogenous locus partially colocalizes with the ELC marker proM2AP. (C) IFA showing the colocalization of the C-terminal tagged Vsp45-HA and N-terminal tagged myc-Stx16. (D) Vsp45-mAID-Ha coimmunoprecipitates with myc-Stx16. (E) Vps45 knockdown leads to a decrease in N-terminal tagged myc-Stx16 protein steady-state levels as shown by the Western blot. Catalase is used as loading control. (F) Plaque assay of TgVps45-mAID-HA and the Tir1 parental strain in the presence or absence of IAA. TgVps45-mAID-HA knockdown but not the parental Tir1 strain displays a severe growth defect in the presence of IAA. (G) TgVps45-mAID-HA knockdown ± IAA. Parasite morphology is grossly affected following depletion of Vps45. Error bars represent the ± SD for three independent experiments. (H) IFA of VspVps45-mAID-HA parasites ± IAA (24 hours). GAP50-GFP and subpellicular microtubules are not formed in the absence of Vps45. GAP50, IMC; GAP45, parasite periphery. (I) IFA of Vsp45-mAID-HA parasites ± IAA (24 hours). Parasites depleted in Vsp45 exhibit an asynchronous block in the IMC formation. GAP50-GFP, IMC marker. (J) IFA of TgVps45-mAID-HA parasites ± IAA (24 hours). Conoid biogenesis is not impaired in Vps45-depleted parasites. Apical cap, AC9-Ty. GAP50, IMC; GAP45, parasite periphery. Scale bars for all IFA = 7 μm.

Vps45 is typically involved in endosome to *trans*-Golgi retrograde transport and vacuolar sorting (43, 44). TgVps45 (TGME49_271060, Table 1) was C-terminally tagged at the endogenous locus with mAID-HA to generate TgVps45-mAID-HA (Fig. S2A). TgVps45-mAID-HA partially colocalizes with GRASP-GFP (Fig. 2A and Fig. S2B) and the ELC marker pro-M2AP (Fig. 2B). In mammalian cells, Vps45 is known to bind to and prevent degradation of Stx16 (45). TgStx16 (TGGT1_247930, Table 1) was N-terminally epitope-tagged at the endogenous locus (myc-TgStx16) in TgVps45-mAID-HA expressing parasites (Fig. S2C), and both proteins were shown to colocalize (Fig. 2C). Moreover, interaction between TgStx16 and TgVps45 was established by coimmunoprecipitation assessed by mass spectrometry (Table S1) and Western blot analysis (Fig. 2D). Importantly, the level of myc-TgStx16 dropped upon TgVps45 depletion as confirmed by Western blot analysis (Fig. 2E).

10.1128/mBio.02092-20.2 FIG S2 (A) PCR demonstrates correct integration of Vsp45-mAID-HA. 9075/9076, 1,100 bp (wild-type locus); 9075/7081, 850 bp. (B) IFA showing the endogenously C-terminally tagged Vsp45 localization. Actin, parasite cytosol. (C) PCR demonstrates correct integration of myc-Stx16 N-terminal tagging. 9499/9500, 600 bp (wild-type locus); 9499/9500, 700 bp (recombinant locus). (D) IAA-induced Vsp45-mAID-HA degradation in intracellular parasites within 2 hours. Catalase, loading control. (E) IFA of Vsp45-mAID-HA parasites ± IAA (24 hours). SAG1, parasite plasma membrane; GAP45, parasite pellicle. (F) IFA of Vsp45-mAID-HA parasites ± IAA (24 hours). Actin, parasite cytosol; GAP45, parasite pellicle. (G) IFA of Vsp45-mAID-HA parasites ± IAA (24 hours). GRASP-GFP, parasite Golgi apparatus; GAP45, parasite pellicle. (H) IFA of Vsp45-mAID-HA parasites ± IAA (24 hours). GRA3, parasite parasitophorous vacuole; GAP45, parasite pellicle. Download FIG S2, PDF file, 0.9 MB. Copyright © 2020 Bisio et al. 2020 Bisio et al. This content is distributed under the terms of the Creative Commons Attribution 4.0 International license.

10.1128/mBio.02092-20.7 TABLE S1 Number of unique spectral counts detected for Vps45 interactors. PL, protein length; TSP, total spectral counts. Download Table S1, DOCX file, 0.02 MB. Copyright © 2020 Bisio et al. 2020 Bisio et al. This content is distributed under the terms of the Creative Commons Attribution 4.0 International license.

**FIG 3:**
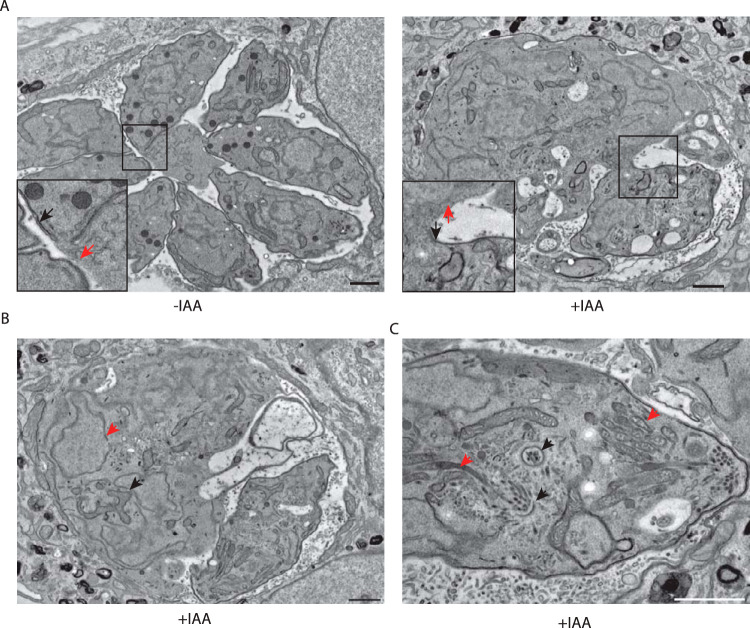
Ultrastructure of TgVps45-depleted parasites showed perturbations in IMC biogenesis. (A) Electron micrographs reveal severe morphological defects in Vps45-iKD parasites associated with a specific lack of newly formed IMC. Regions of the plasma membrane that lack association with the IMC are indicated with red arrows, while mature pellicle is indicated with black arrows. (B) Electron microscopy confirms correct karyokinesis (red arrowhead) and normal mitochondrion (black arrowhead). (C) Electron microscopy reveals correct conoid biogenesis (black arrowheads) and rhoptry docking (red arrowheads) in the absence of Vps45. Scale bars = 1 μm.

Depletion of TgVps45 in the presence of IAA (Fig. S2D) led to a severe phenotype by plaque assay (Fig. 2F), which is accompanied by a loss in parasite structural integrity (Fig. 2G and Fig. S2E). Although parasites depleted in TgVps45 still possess an intact plasma membrane (Fig. S2E and F), they display a severe defect in IMC biogenesis and, likely as a consequence of this, a defect in subpellicular microtubule assembly (Fig. 2H). Importantly, a similar phenotype has been associated with the depletion of TgStx16 in T. gondii (46). This defect was observed at every step during IMC formation since parasites treated with IAA were sequestered asynchronously during daughter cell formation as demonstrated by GAP50-GFP detection (Fig. 2I). Parasites lacking TgVps45 manage to accomplish karyokinesis with preservation of the nuclear associated Golgi (Fig. S2G) and proper centrosome positioning. This confirms previous data showing that there is no checkpoint in the cell cycle of T. gondii to complete IMC formation (47). Moreover, the conoid is assembled in the absence of TgVps45 (Fig. 2J), and rhoptries are still attached to the tip of the parasites (Fig. 3B), indicating that apical organelle biogenesis is disconnected form IMC biogenesis. Finally, the secretion of dense granules was not impaired in the absence of Vps45 as shown by accumulation of GRA3 at the PVM (Fig. S2H) and proper formation of the nanotubular network (48) (Fig. 3A). The abnormalities in IMC formation observed by indirect immunofluorescence analysis (IFA) were confirmed at the level of electron microscopy (Fig. 3A). The IMC of the parental parasite subjected to TgVps45 depletion remained intact, while the material resulting from multiple rounds of karyokinesis and newly formed conoid and apical organelles accumulated in the cytoplasm (Fig. 3B and C).

### Vps45 function in IMC biogenesis is also conserved in Plasmodium falciparum.

**FIG 4:**
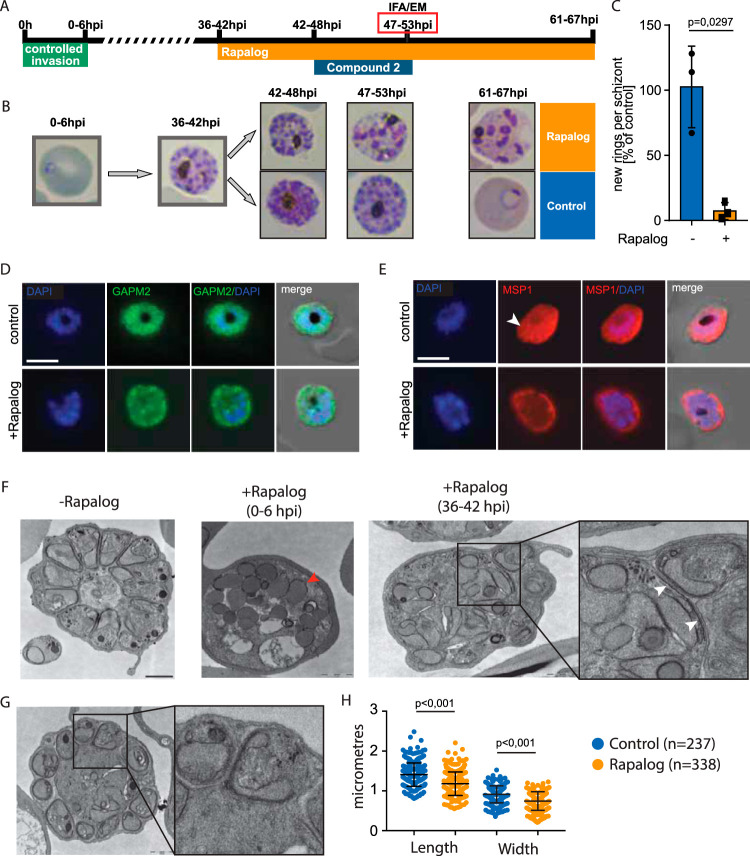
*Pf*Vps45 plays a crucial role in IMC biogenesis. (A) Schematic illustration of the experiment to conditionally inactivate PfVPS45 in schizonts. (B) Giemsa smears demonstrate that parasites lacking functional PfVps45 are arrested at the schizont stage upon induction of knock sideways (rapalog) at 36 to 42 hpi. (C) Quantification of newly formed rings per schizont after the removal of compound 2. Parasites ± rapalog (25 hours). Bars show the SD for three independent experiments. (D) IFA of *Pf*Vps45 KS parasites ± rapalog (11 hours). IMC biogenesis is inhibited in parasites when Vps45 is mis-localized. GAPM2, parasite IMC; DAPI, nuclei. Scale bar = 5 μm. (E) IFA of *Pf*Vps45 KS parasites ± rapalog (11 hours). Schizont plasma membrane fails to engulf merozoites when Vps45 is mis-localized. MSP1, parasite plasma membrane; DAPI, nuclei. An arrowhead points to fully formed merozoites with engulfed plasma membrane in wild-type conditions. Scale bar = 5 μm. (F) Electron micrographs (TEM) of ± rapalog-treated Vps45 KS parasites (47 to 53 hpi). Early treatment at the ring stage (0 to 6 hpi) with rapalog leads to arrest of the parasites in the trophozoite stage. Later treatment at the early schizont stage (36 to 42 hpi) leads to a partial IMC biogenesis defect. IMC biogenesis initiates but failed to elongate (right inset). White arrowheads show IMC, while the red arrowhead shows endocytic vesicles. Scale bars = 1 μm. (G) Schizonts formed in the presence of rapalog possess an enlarged residual body and smaller merozoites (right inset). Scale bar = 1 μm. (H) Quantification of the size of merozoites formed in the presence or absence of rapalog. Bars show the SD.

In P. falciparum, PfVps45 was implicated in the transport of host cell cytosol-containing endosomes to the parasite food vacuole (15). Here, we used the corresponding cell line to analyze PfVPS45. As previously reported (15), after conditional mis-localization of PfVps45 in early stages of the development of P. falciparum in red blood cells (RBCs), parasites became congested with vesicles of endocytosed material and failed to progress to schizonts (Fig. S3A), hampering examination of the role of PfVps45 in IMC biogenesis. To overcome this and test whether PfVPS45 might also have a role in IMC biogenesis and cytokinesis, we selectively inactivated PfVPS45 in schizont-stage parasites and monitored final schizont development (Fig. 4A). Induction of the mis-localization of Vps45 using rapalog (Rapa) in 36- to 42-hour postinvasion schizonts strongly inhibited schizogony (Fig. 4A and B and Fig. S3B). This arrest in intraerythrocytic development prevents egress, reinvasion and formation of new rings (Fig. 4C). This defect was associated with a defect in IMC formation (Fig. 4D and Fig. S3C) and with a failed invagination of the plasma membrane, a process that in wild-type (WT) cells leads to the formation of the daughter merozoites (Fig. 4E and Fig. S3C). Similar to the phenotypes observed in T. gondii, rhoptries and micronemes are mostly unaffected in malaria parasites when PfVPS45 is inactivated late in the cycle (Fig. S4A and B). Electron microscopy analysis revealed that upon depletion of PfVps45, the IMC was partially formed but failed to engulf cytoplasmic content to form the merozoites (Fig. 4F; +Rapalog [36 to 42 hours postinfection (hpi)]), and this clearly contrasted with the vesicle congestion phenotype observed when PfVPS45 was inactivated early in the cycle (Fig. 4F; +Rapalog [0 to 6 hpi]). Not all parasites showed a cytokinesis defect, and some led to the formation of merozoites (Fig. 4G). Some of the merozoites formed were significantly smaller than the wild type (Fig. 4H), resembling a defect of other proteins associated with correct IMC formation (49–51). Moreover, the size of the residual body was increased in schizonts formed in the absence of PfVps45 (Fig. 4G). Accumulation of host cell cytosol-filled endocytic vesicles was also observed in schizonts, indicating that endocytosis is active even at a very late stage of the development of P. falciparum parasites in red blood cells.

10.1128/mBio.02092-20.3 FIG S3 (A) Schematic overview of the experiment and Giemsa staining demonstrate that parasites lacking functional PfVsp45 are arrested at the trophozoite stage upon rapalog treatment at 0 to 6 hpi. (B) Giemsa staining demonstrates that parasites lacking functional PfVsp45 are arrested at the schizont stage upon rapalog treatment at 36 to 48 hpi. (C and D) IFA of *Pf*Vsp45 KS parasites ± rapalog (24 hours). IMC biogenesis is inhibited when Vsp45 is mis-localized. GAPM2, parasite IMC. The schizont plasma membrane fails to engulf merozoites when Vsp45 is mis-localized. MSP1, parasite plasma membrane. Download FIG S3, PDF file, 0.1 MB. Copyright © 2020 Bisio et al. 2020 Bisio et al. This content is distributed under the terms of the Creative Commons Attribution 4.0 International license.

10.1128/mBio.02092-20.4 FIG S4 (A) IFA of *Pf*Vsp45 KS parasites ± rapalog (24 hours). MSP1, parasite plasma membrane; AMA1, parasite micronemes. (B) IFA of *Pf*Vsp45 KS parasites ± rapalog (24 hours). MSP1, parasite plasma membrane; RAP1, parasite rhoptries. Download FIG S4, PDF file, 0.1 MB. Copyright © 2020 Bisio et al. 2020 Bisio et al. This content is distributed under the terms of the Creative Commons Attribution 4.0 International license.

### Toxoplasma gondii Vps45 plays a role in ELC and VAC homeostasis and trafficking of host cell protein cargo for digestion.

**FIG 5:**
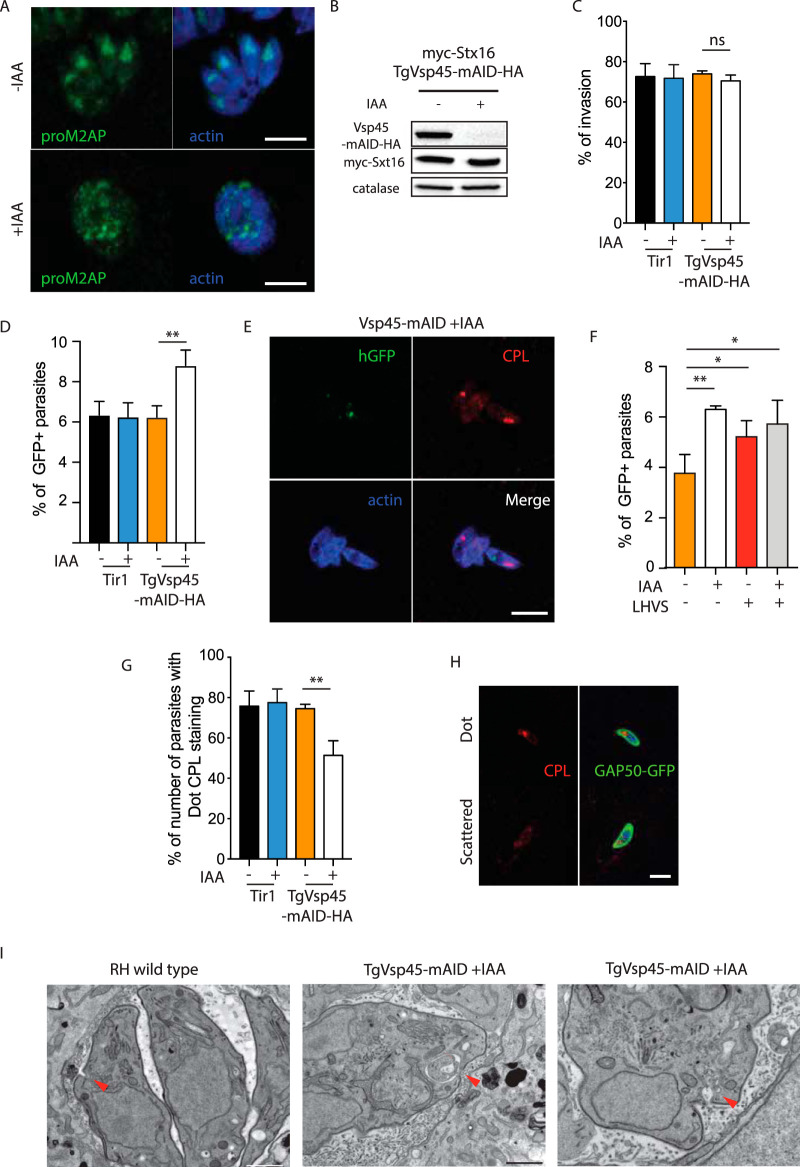
TgVps45 is essential for ingestion and digestion of cytosolic host proteins. (A) IFA of TgVps45-mAID-HA parasites ± IAA (24 hours). ELC homeostasis is grossly affected in the absence of TgVps45. proM2AP, ELC; actin, parasite cytosol. (B) IAA-induced TgVsp45-mAID-HA degradation in extracellular parasites within 2 hours. Catalase, loading control. (C) Parasites lacking TgVps45 are not impaired in invasion. Error bars represent the ± SD for three independent experiments. (D) Quantification of ingestion of host cytosolic GFP 7 minutes postinfection in WT or Vps45-mAID-HA knockdown ± IAA parasites. The percentages of GFP-positive tachyzoites from three independent experiments are shown. (E) IFA of Vps45-mAID-HA parasites + IAA (30 minutes postinfection of GFP-positive host cells). Colocalization of ingested GFP with the VAC was rarely observed. CPL, VAC; actin, parasite cytosol. (F) Quantification of ingestion of host cytosolic GFP 30 minutes postinfection in Vps45-mAID-HA knockdown ± IAA parasites ± LHVS. The percentages of GFP-positive tachyzoites from three independent experiments are shown. (G to H) The VAC morphology is dynamic and can appear as a single dot or a scattered pattern. The morphology of VAC is affected following depletion of TgVps45 (G). Error bars represent the ± SD for three independent experiments. Representative images are shown in panel H. CPL, VAC; GAP50, parasite IMC. (I) Electron microscopy reveals an enlarged micropore in Vps45 conditional knockdown (cKD) parasites. Arrowhead, micropore. Scale bars = 1 μm. Scale bars for all IFA = 7 μm.

Since the efficient digestion of host cell cytosolic proteins is not essential for T. gondii tachyzoites (20), we reasoned that the defect of TgVps45 in IMC formation might mask a conserved function of TgVps45 for endocytosis and/or trafficking of host cell protein cargo for digestion. To assess the potential role of TgVps45 in endocytosis, we first analyzed the importance of this protein for the ELC. Depletion of Vps45 by IAA led to gross morphological changes in the ELC compartment, which adopted a dispersed punctuate pattern (Fig. 5A). To specifically assess protein ingestion, extracellular parasites were treated with IAA for 2 hours, which leads to a complete degradation of the TgVps45 but not its interacting partner TgStx16 (Fig. 5B). Parasites lacking Vps45 were capable of invading host cells normally (Fig. 5C). In order to assess endocytosis, we performed an assay that scored the amount of protein ingested from host cells by using HEK cells expressing cytosolic green fluorescent protein (GFP) (20). Parasites lacking Vps45 quickly incorporated host GFP 7 minutes after infection and showed an increased intracellular GFP staining compared to wild-type parasites (Fig. 5D). These data are consistent with an inability to transport endocytic vesicles to the VAC and digest internalized host proteins or an increase in endocytosis. Interestingly, we rarely observed colocalization of GFP ingested protein and the VAC marker cathepsin L (CPL) upon 30 minutes of infection (Fig. 5E) in parasites downregulated in TgVps45, indicating that endosomes containing ingested proteins are incapable of fusing with the VAC compartment, akin to the endocytic vesicle transport phenotype observed after inactivation of VPS45 in P. falciparum (15). This indicates a role of TgVps45 in digestion of host proteins potentially by regulating the fusion of endosome/ELC-derived vesicles with the VAC. In order to further dissect a role of Vps45 in protein trafficking for digestion, parasites were incubated with morpholinurea-leucine-homophenylalanine-vinyl phenyl sulfone (LHVS), an inhibitor of CPL, which renders the parasites incapable of digesting ingested proteins (20, 21). Concordantly with a role of Vps45 in endosome fusion with the VAC, GFP-positive parasites did not increase in the presence of LHVS upon treatment with IAA (Fig. 5F). Moreover, while wild-type parasites typically displayed a single VAC with strong staining of CPL (25) 30 minutes postinvasion of a new host cell, parasites lacking TgVps45 showed a scattered CPL marker (Fig. 5H), suggesting a dysregulation in VAC homeostasis, in contrast to what was observed when Vps45 was inactivated in P. falciparum blood stages which led to an accumulation of endocytic vesicles on the way to the food vacuole without disrupting the food vacuole (15). Interestingly, in the few vacuoles where we could observe the micropore in parasites depleted of TgVps45, electron microscopy images revealed morphological abnormalities at the level of this structure (Fig. 5F), which is presumably involved in endocytosis (17). While wild-type parasites presented a small, cup-shaped depression, Vps45-depleted parasites showed an enlarged and deformed micropore filled with membranous material (Fig. 5F). This structure was not observed in all vacuoles depleted in Vps45, and the relevance for the phenotype of Vps45 remains to be fully addressed.

### Several SNARE proteins define trafficking through the ELC.

**FIG 6:**
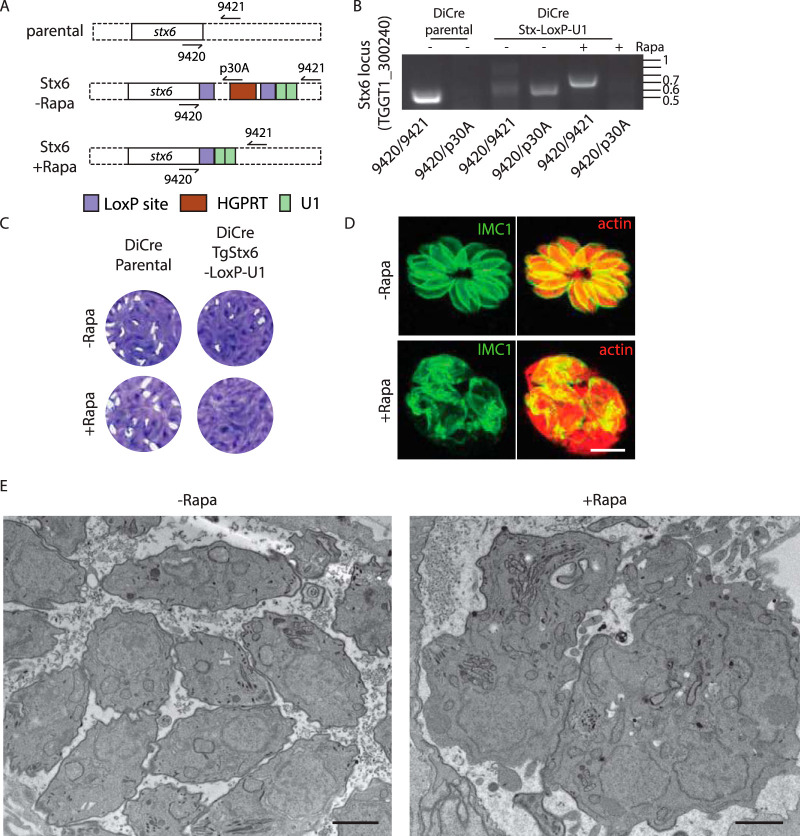
TgStx6 is essential for ELC trafficking. (A) Schematic representation of the strategy used to generate the recombinant TgStx6 strain. (B) PCR demonstrates correct integration and excision upon rapamycin treatment. 9323/9324, 459 bp (wild-type locus); 9323/p30A, 531 bp; 9323/9324, 674 bp (Cre excised locus). (C) Stx6-LoxP-U1 cKD but not the parental DiCre strain displays a severe growth defect in the presence of rapamycin. (D) IFA of TgStx6-LoxP-U1 cKD ± rapamycin. Parasite morphology is grossly affected following depletion of TgStx6. IMC1, inner membrane complex; actin, parasite cytosol. Scale bars = 7 μm. (E) Electron microscopy reveals gross morphological defects in Stx6 cKD parasites associated with a specific lack of newly formed IMC. Scale bars = 1 μm.

The targeting determinants of the trafficking vesicles depend on SNARE proteins interacting in a combinatorial manner with tethering factors (2). In mammalian cells, Vps45 forms a complex with Stx6 (TGGT1_300240, TgStx6), TgStx16 (TGGT1_278160), and TgVti1 (putatively TGGT1_242080 or TGGT1_278160) to mediate fusion of early endosome vesicles with the TGN (52, 53). TgStx6 was previously shown to localize to the trans-Golgi apparatus (54) and was detected by coimmunoprecipitation with TgVps45 by mass spectrometry (Table S1). To determine if such a complex might be also formed and fulfil similar functions in apicomplexans, we engineered the endogenous locus of TgStx6 using the LoxP/U1 RNA destabilization strategy in a DiCre-expressing strain (55–57). Correct integration, replacement of the 3′ untranscribed region (UTR), and rapamycin-induced DiCre-mediated excision of TgStx6 were analyzed by PCR (Fig. 6A and B). As previously reported with dominant negative mutants of Stx6 (54), downregulation of TgStx6 compromised the lytic cycle (Fig. 6C), impacting IMC biogenesis and parasite integrity (Fig. 6D and E) and displaying a similar phenotype as observed upon TgVps45 depletion. Previous work reported that TgStx6 impacts the formation of the IMC and ELC homeostasis (54), mimicking the phenotype associated with the depletion of TgVps45 and suggesting the possibility that TgStx6 could work in concerted action with TgVps45 and TgStx16 (at least for some of their functions) also in T. gondii.

**FIG 7:**
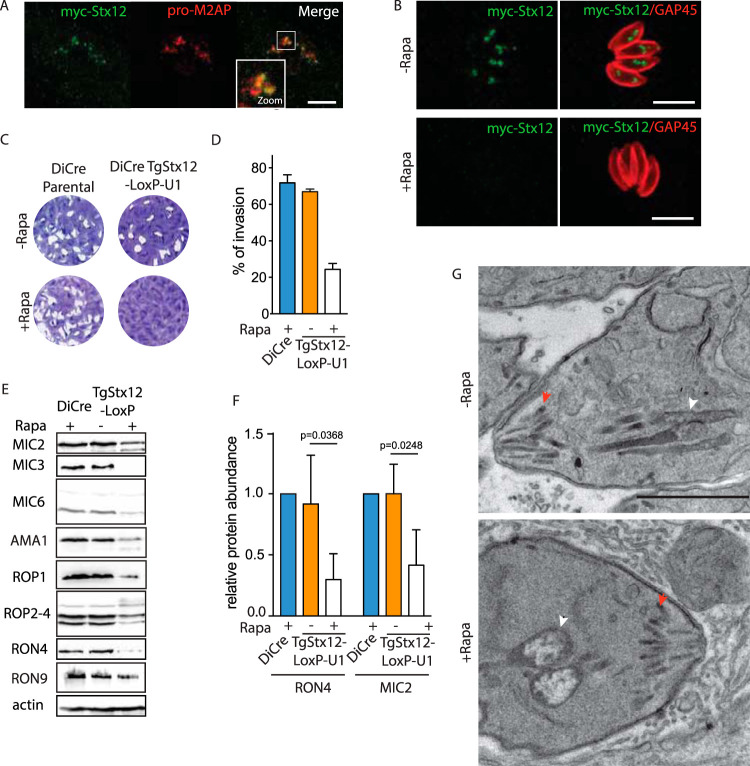
TgStx12 is essential for microneme and rhoptry function. (A) Indirect immunofluorescence assay (IFA) showing the endogenously N-terminally tagged Stx12 localized to the ELC. ProM2AP, parasite ELC marker. (B) Rapamycin-induced Stx12 downregulation in intracellular parasites within 24 hours. GAP45, parasite pellicle. (C) The Stx12-LoxP-U1 knockdown but not the parental DiCre strain displays a severe growth defect in the presence of rapamycin. (D) Parasites lacking Stx12 show a significantly impaired invasion capacity. Error bars represent the ± SD for three independent experiments. (E) The content of microneme and rhoptry localized proteins is reduced upon depletion of Stx12. Catalase, loading control. (F) The quantification of RON4 and MIC2 are shown. Error bars represent the ± SD for three independent experiments. (G) Electron microscopy reveals no gross morphological defects of micronemes and mild abnormalities of rhoptries in parasites depleted in Stx12. Micronemes and rhoptries are indicated with red and white arrowheads, respectively. Scale bars = 1 μm. Scale bars for all IFA = 7 μm.

Stx12 (TGGT1_204060, TgStx12) was previously shown to regulate several steps of fusion in the prevacuolar compartment in yeast (58, 59). Myc-TgStx12 was shown to colocalize with the ELC marker pro-M2AP (Fig. 7A) but not with the Golgi marker (GRASP-GFP) (Fig. S5A), VAC marker (CPL) (Fig. S5B), apicoplast marker (CPN60) (Fig. S5C), or rhoptry neck marker (RON4) (Fig. S5D). Depletion of TgStx12 using the same strategy (Fig. 7B, Fig. S5E and F) led to complete impairment in the parasite lytic cycle (Fig. 7C). This defect was associated not with any intracellular growth defect (Fig. S5G) or alteration in egress (Fig. S5H), but with an impairment in invasion (Fig. 7D). Freshly egressed parasites depleted in TgStx12 showed no major alteration in microneme secretion (Fig. S5I) or rhoptry discharge (Fig. S5J and S5K) upon 48 hours of rapamycin treatment. In contrast, the total amount of mature microneme and rhoptry proteins processed by TgASP3 (13) was significantly decreased in the absence of TgStx12 (Fig. 7E and F). This small amount of protein produced was correctly delivered to the respective secretory organelles in the case of proteins such as MIC2 or ROP2-4 (Fig. S6A and C) but clearly mis-localized in cases of MIC6 or RON4 (Fig. S6B and D). The secretory organelles were correctly positioned as visualized by organelle surface markers (APH [(60] and ARO [6]) after 48 hours of rapamycin treatment (Fig. S6E and F). Moreover, after 72 hours of rapamycin treatment, no major abnormalities in microneme ultrastructure were observed, although the rhoptries showed an enlarged bulb (Fig. 7G). Importantly, moving junction formation was decreased in TgStx12-depleted parasites to similar levels as invasion rates. In the few occasions the moving junction was seen upon depletion of Stx12, it appeared normal by IFA compared to wild-type conditions (Fig. S5L). These data suggest that despite normal levels of rhoptry discharge and microneme exocytosis (Fig. S5I to K), lower levels of the protein content of these organelles might result in a lower efficiency of moving junction formation.

10.1128/mBio.02092-20.5 FIG S5 (A to D) Indirect immunofluorescence assay (IFA) showing that the endogenously N-terminally tagged Stx12 do not colocalize with the Golgi apparatus (A), VAC compartment (B), apicoplast (C), or rhoptry (D). GRASP-GFP, parasite Golgi apparatus; CPL, VAC; Cpn60, apicoplast; RON4, rhoptry neck. (E) Schematic representation of the strategy used to generate the recombinant Stx12 strain. (F) PCR demonstrates correct integration and excision upon rapamycin treatment. 9420/9421, 768 bp (wild-type locus); 9420/p30A, 794 bp; 9420/9421, 983 bp (Cre excised locus). (G to H) Parasites lacking Stx12 are not impaired in intracellular replication (G) or egress (H). Error bars represent the ± SD from three independent experiments. (I) EtOH (2%) stimulation of microneme secretion is not affected in parasites lacking Stx12. GRA1, control for parasite viability. Catalase was used as the loading control and lysis control. SUB1 processed and unprocessed MIC2 are indicated by red and black triangles, respectively. (J to K) Rhoptry discharge is not affected in parasites lacking Stx12 as shown by phosphorylation of Stat6 (J) or evacuole formation (K). (L) Representative IFA image of parasites that manage to form a moving junction. CPL, VAC; GAP45, parasite pellicle; RON4, moving junction. Error bars represent the ± SD for three independent experiments. Download FIG S5, PDF file, 0.8 MB. Copyright © 2020 Bisio et al. 2020 Bisio et al. This content is distributed under the terms of the Creative Commons Attribution 4.0 International license.

10.1128/mBio.02092-20.6 FIG S6 (A to F) IFA of Stx12-LoxP-U1 parasites ± rapamycin (24 pretreatment + 24 hours). MIC2 and MIC6, microneme proteins; ROP2-4, rhoptry bulb protein; RON4, rhoptry neck; APH, microneme surface marker; GAP45, marker of the parasite pellicle; ARO, rhoptry surface marker; actin, parasite cytosol. Download FIG S6, PDF file, 0.1 MB. Copyright © 2020 Bisio et al. 2020 Bisio et al. This content is distributed under the terms of the Creative Commons Attribution 4.0 International license.

**FIG 8:**
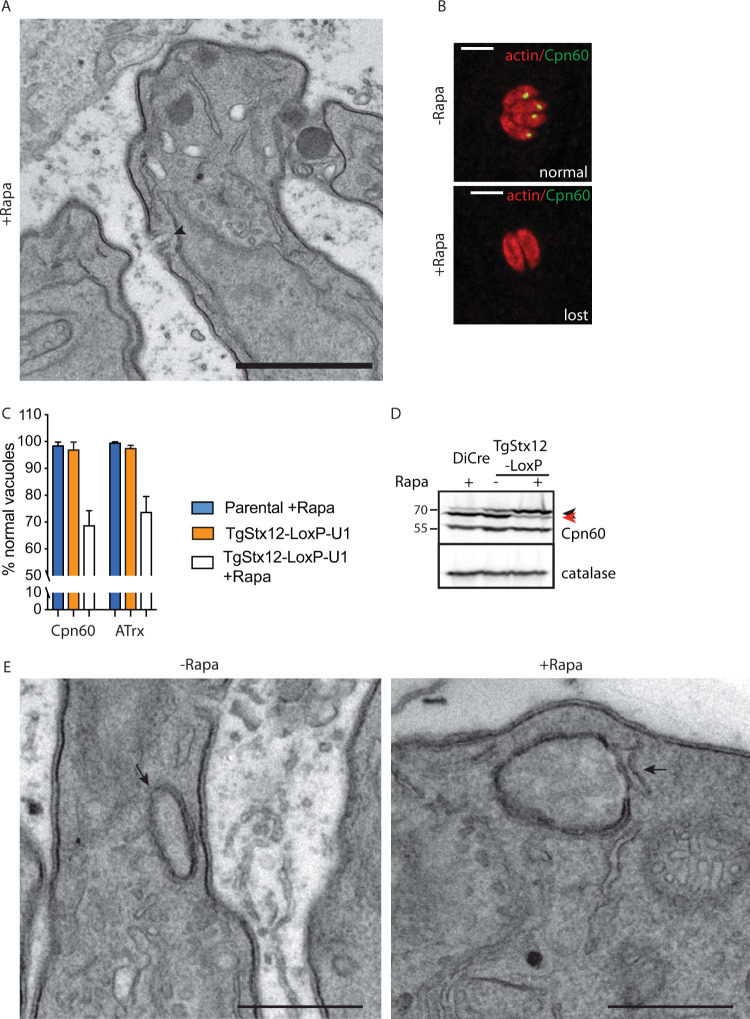
TgStx12 is essential for protein transport into the apicoplast. (A) Electron microscopy reveals no gross morphological defects of the micropore (indicated with an arrowhead) in parasites depleted in Stx12. (B) Apicoplast is shown to be lost in a subpopulation of Stx12 cKD parasites upon 72 hours of treatment with rapamycin. Representative images are shown. Cpn60 or ATrx were used as apicoplast markers. Scale bars = 7 μm. (C) Quantification of apicoplast upon depletion of Stx12 for 72 hours with rapamycin. Error bars represent the ± SD for three independent experiments. (D) Pro-Cpn60 (marked with a black arrowhead) accumulates upon depletion of Stx12 for 72 hours with rapamycin. Processed Cpn60 is marked with a read arrowhead. (E) Electron microscopy reveals no gross morphological defects of the apicoplast (indicated with an arrow) in parasites depleted in Stx12. Scale bars in A and E = 1 μm.

The severe invasion defect observed on TgStx12 depletion and the slow kinetics of the knockdown system prevented assessment of TgStx12’s implication in endocytosis. Micropore morphology, however, was shown to be normal in parasites depleted in TgStx12 (Fig. 8A). Interestingly, the stromal apicoplast protein CPN60 and the peripheral apicoplast protein ATrx were not detectable in ∼30% of the population of the TgStx12-depleted parasites 72 hours after rapamycin addition (Fig. 8B and C). Of relevance, Western blot analysis of parasites depleted in TgStx12 after 48 hours of rapamycin treatment revealed a significant accumulation of pro-CPN60 (Fig. 8D). CPN60 is a nuclear-encoded apicoplast protein targeted to the plastid via its bipartite signal which is cleaved during import (61), yet the morphology of the apicoplast was not affected by downregulation of TgStx12 (Fig. 8E).

## DISCUSSION

Vesicular trafficking plays central roles in eukaryotic cells, participating in the genesis of organelles, communication between organelles, the secretion and internalization of diverse cargo out of and into the cell, and membrane and cargo recycling processes. Studies have addressed the roles of Rab GTPases (11) and phospholipids (15, 60) in the trafficking and vesicle fusion in apicomplexans, yet the SNARE proteins have been largely neglected. Interestingly, despite the complexity of organelles and structures present in apicomplexan parasites and the necessity of three to four different types of SNARE proteins to trigger membrane fusion, only a small subset of SNARE proteins is found in these parasites. The genome of T. gondii encodes 7 R-type SNARE, 5 Qa SNARES, 7 Qb SNARES, 5 Qc SNARES, and 1 Qbc SNARE (Table 1). Defined specificity by combinatorial interactions of SNARE proteins allow the number of “identifiers” available for vesicle trafficking to increase (2). For instance, in mammalian cells (53, 62), complexes involving Vps45, Stx16, and Stx6 regulate early endosome-to-TGN fusion, while a Vps45, Stx16, and Stx10 complex regulates late endosome-to-TGN fusion events. The degree of conservation of these pathways remains to be fully addressed in apicomplexan parasites, though our data suggest a certain degree of conservation (Table S1).

**FIG 9:**
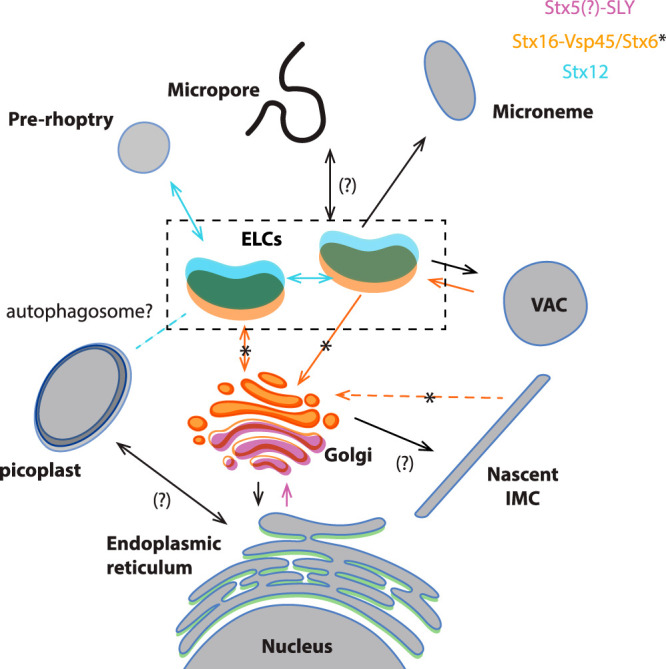
Model of vesicle trafficking in apicomplexan parasites. The model presented in this figure represents a combined adaptation of the trafficking models presented in references 11 and 21. Organelles are colored depending on the localization of proteins studied here. Putative fusion events involving these proteins are indicated with colors in the arrows. The directionality of the transport has been deducted considering that q-SNARES usually localize on the target membranes (2, 3). Proteins transported through the secretory pathway are synthesized in the rough ER. Vesicles shed from the ER are transported and fuse with the cis-Golgi, likely through the action of SLY1. Stx5 is likely an interactor of SLY1, as demonstrated in model organisms (64), mediating the fusion of these vesicles. The IMC is produced from the trans-Golgi compartment, while recycling of trafficking molecules needed for IMC biogenesis likely involves Vsp45 (likely mediated by its interacting partner Stx16) and Stx6. Transport between the ELC to the trans-Golgi involves Vsp45, likely its interacting partner Stx16, and Stx6. Endocytosis is proposed to occur in the micropore or the cytostome (17–19) and transported into subcompartments of the ELC (21). SNARE proteins involved in this process are unknown. Endocytosis and VAC-mediated digestion of internalized host proteins involve Vsp45 and likely Stx16. Microneme and rhoptry proteins are transported to the proper compartment in a temporally regulated manner (21) involving the action of Stx12. Apicoplast transport from the ER is accomplished by unknown SNARE proteins. Interestingly, homeostasis of the apicoplast depends on the ELC compartment and Stx12. A putative role of auto-phagocytosis in this process remains to be addressed.

SNARE proteins involved in trafficking of secretory proteins are evolutionarily conserved. Considering that Q-SNAREs typically localize to target membranes (63), we propose the following working model for intracellular trafficking in T. gondii (Fig. 9), representative of a conservative interpretation of the data. We show here that in T. gondii, trafficking from ER to Golgi likely depends on TgSLY1, a Sec1/Munc18 protein known to bind to Stx5 (putatively TGGT1_226600, TgStx5) (64) (Fig. 9). Interestingly, depletion of TgSLY1 results in a complete inhibition of intracellular growth, in contrast to the depletion of TgVps45. These differences likely reflect the impact of SLY1 in the trafficking between the ER and the Golgi apparatus, which are the hub of the secretory pathway. For example, TgSLY1-depleted parasites are likely unable to expose protein essential for nutrient acquisition (i.e., plasma membrane transporters or dense granule proteins). In addition, a partial impairment of centrosome positioning and duplication due to the lack of a functional Golgi (65) might contribute to the TgSLY1 phenotype, causing an arrest of the cell cycle due to a checkpoint failure (47).

The ELC is an important platform that receives and dispatches vesicles from the Golgi apparatus, micronemes, prorhoptries, and VAC (11). We identified TgVps45, putatively in action with TgStx16 and TgStx6 (54), and TgStx12 to be involved in the selection and transport of some of these cargo vesicles (Fig. 9).

Endocytosis in apicomplexan parasites has been recently linked to artemisinin (66) and chloroquine (67–69) resistance. Internalization is supposed to occur through particular structures such as the micropore (17) or cytostome (19) and might depend on the intravacuolar network (IVN) (20). Interestingly, TgVps45-depleted parasites showed in a few cases an enlarged micropore filled with membranous material with similar electron density to that of the IVN, but further experiments need to be performed to confirm the nature of this material. Once internalized, early endocytic vesicles are hallmarked by phosphatidylinositol 3-phosphate [PI_(3)_P] (70), including in *Plasmodium* parasites (15, 71). Interestingly, likely resembling its evolutionary origin (37), PI_(3)_P seems to be critically involved in protein trafficking to the apicoplast (71, 72). While the importance of endocytosis in malaria parasites is obvious (19), access to host proteins by T. gondii tachyzoites *in vitro* appears less relevant. In contrast, the latent encysted stage of T. gondii, bradyzoites, is critically depend on the VAC function for persistence (14). Endocytosis has also been observed in extracellular T. gondii and suggested to be associated with parasite motility (73). Here, we show that vesicular fusion with the VAC, and possibly endocytosis, depend on TgVps45, similarly to what was previously shown for P. falciparum (15).

The IMC presumably arises from the Golgi, and our data indicate that recycling of the biosynthetic machinery (which includes Rab11B [74] and unknown SNARE proteins) either occurs using the same Q-SNAREs (Vps45-Stx16 complex [46] and Stx6 [54]) as ELC-to-Golgi transport or involves the ELC (54). This feature is evolutionarily conserved in T. gondii and P. falciparum and hence likely also across the Apicomplexa phylum.

Of relevance, TgStx12 is involved in the efficient trafficking of mature proteins to micronemes and rhoptries. The proteins likely unable to properly traffic through the ELC are degraded or mis-localized. No accumulation of the proproteins prior to their cleavage by the ELC resident protease TgASP3 (13), was observed.

Direct evidence for vesicle-mediated trafficking of apicoplast-resident nuclear-encoded proteins is lacking (75). Here, we show that the apicoplast function depends on TgStx12 but not on TgSLY1. This might imply that TgStx12 is associated with the ER-to-apicoplast trafficking of proteins. Importantly, the lack of colocalization between myc-TgStx12 with an apicoplast marker or ER staining does not favor a direct role of TgStx12 in the ER-to-apicoplast trafficking. Another possibility is an association of TgStx12 with autophagy, which has been previously hypothesized to be directly linked to the apicoplast as a putative phagopore assembly site (76). This might implicate autophagy as an important player in apicoplast homeostasis. Finally, the effect of TgStx12 on the trafficking of nuclear-encoded apicoplast resident proteins might be an indirect effect caused by a traffic jam not generated in the absence of TgSLY1 due to rapid loss of parasite viability.

Another important observation arising from this study is that trafficking pathways in apicomplexans appear to be conserved at least to a certain level, and some of the differences observed between *Plasmodium* and *Toxoplasma* might represent adjustments for their mode of division, organelle biogenesis, and metabolic needs. Shown here, both TgVps45 and PfVps45 are involved in IMC biogenesis and endosomal transport of internalized host cell proteins. Discrepancies in the phenotype (15, 46) arise from the late biogenesis of daughter cells in the malaria parasite and the limited role of host protein degradation as a source of nutrients in the tachyzoite stage of T. gondii.

Taken together, we identified SNARE and Sec1/Munc18 proteins that generate the ZIP code necessary for vesicular trafficking from several organelles, such as the ER, Golgi, and through the ELC, an intersecting point of endocytosis and exocytosis, impacting the various unique compartments found in apicomplexans.

## MATERIALS AND METHODS

### Antibodies and reagents.

Primary antibodies used in this study include mouse anti-Ty tag BB2, mouse anti-Myc 9E10, rabbit anti-hemagglutinin (HA) (sigma), mouse ac-tubulin (sc-23950, Santa Cruz Biotechnology), mouse anti-MIC2 (gift from J. F. Dubremetz), mouse anti-SAG1 (T4-1E5), mouse anti-actin (77), mouse anti-GRA1 (gift from J. F. Dubremetz), rabbit anti-IMC1 (78), rabbit anti-GAP45, anti-catalase (79), anti-MSP1, polyclonal (polyclonal antiserum), anti-GAPM2 (80), anti-RAP1 (81), anti-AMA1 (MRA-481A), and proM2AP/CPL (gift from V. B. Carruthers).

Secondary antibodies Alexa Fluor 488 and Alexa Fluor 594-conjugated goat anti-mouse/rabbit/rat antibodies (Molecular Probes) were used for immunofluorescence analysis, and horseradish peroxidase (HRP)-conjugated goat anti-mouse/rabbit/mouse antibodies (Molecular Probes) were used for Western blot analysis.

Compound 2 was obtained from Mike Blackman (A/C heterodimerizer AP21967 [rapalog]; Clontech; catalog number 635055).

### Parasite culture and transfection.

Tachyzoites of the T. gondii RH strains lacking HXGPRT and KU80 and expressing Tir1 (gift from David Sibley) or DiCre (gift from Moritz Treeck) were grown in confluent HFF monolayers maintained in Dulbecco’s modified Eagle’s medium (DMEM; Gibco) supplemented with 10% fetal calf serum (FCS), 2 mM glutamine, and 25 mg/ml gentamicin. Parasites were transfected and selected as described previously (82).

P. falciparum parasites were grown at 37°C in RPMI 1640 complete medium containing 0.5% Albumax (Life Technologies) and erythrocytes (blood group 0+; transfusion blood; Universitatsklinikum Hamburg-Eppendorf) at a hematocrit of 5% and cultured according to standard methods (83).

### Cloning of DNA constructs.

All primers used in this study are listed in Table S2. Auxin-inducible degradation of SLY (TGGT1_213072) and Vps45 (TGGT1_271060) was generated using a PCR fragment encoding the mAID-HA and the HXGPRT cassette produced using the KOD DNA polymerase (Novagen, Merck) with the vector pTUB1:YFP-mAID-3HA as the template and the primers 8927/8928 and 8930/8931, which also carry 30 bp homology with the 3′ end of SLY or Vps45. A specific single guide RNA (sgRNA) was generated to introduce a double-stranded break at the 3′ end of the genes (primers used to generate the guide included 8926 and 8929/4883).

10.1128/mBio.02092-20.8 TABLE S2 Primers used in this study. Download Table S2, DOCX file, 0.01 MB. Copyright © 2020 Bisio et al. 2020 Bisio et al. This content is distributed under the terms of the Creative Commons Attribution 4.0 International license.

U1-mediated knockdown of the Stx6 and Stx12 genes was generated using a PCR fragment encoding the loxP flanked 3′ UTR of SAG1 and the HXGPRT selection cassette followed by four U1 recognition sites using the KOD DNA polymerase (Novagen, Merck). pLIC-HA-FLAG-(3′UTRSAG1-pDHFR-HXGPRT-5′UTRDHFR)-flox-4xU1 was used as the template vector and amplified using the primers 9304/9305 and 9307/9308 for modification of the Stx6 and Stx12 loci, respectively. A specific sgRNA was generated to introduce a double-stranded break at the 3′ end of the genes (primers used to generate the guide included 9303 and 9306/4883).

Ty tagging of the AP2 alpha subunit was generated using a PCR fragment encoding the 2xTy tag and HXGPRT selection cassette using the KOD DNA polymerase (Novagen, Merck). Plinker-2Ty-HXGPRT was used as the template vector and amplified using the primers 9442/9443. A specific sgRNA was generated to introduce a double-stranded break at the 3′ end of the genes (primers used to generate the guide included 9441/4883).

N-terminal myc tagging of the Stx16 was generated using a PCR fragment encoding the 4xmyc tags using the KOD DNA polymerase (Novagen, Merck). The 4xmyc sequence was ordered as a synthetic gBlock fragment (IDT DNA) and was used as the template to be amplified using the primers 9425/9426. A specific sgRNA was generated to introduce a double-stranded break at the 5′ end of the genes (primers used to generate the guide included 9424/4883).

### Knock sideways with VPS45 in P. falciparum schizonts.

To gain a synchronous culture with a narrow time window, parasites of the PfVps45-2xFKBP-GFPint + 1xNLS-FRB-mCh cell line (15) were purified using 60% Percoll. These purified parasites were added to uninfected erythrocytes and permitted to invade for 6 hours under culture conditions followed by synchronization with 5% sorbitol. The resulting freshly invaded 0 to 6-hour-old ring-stage parasites were further cultured until they reached the early schizont stage (36 to 42 hours p.i). At this point the culture was split into two parts, and the knock-sideways of the Vps45 protein was induced by adding the heterodimerizer rapalog (Clontech) to a final concentration of 250 nM in one part while keeping the other as an untreated control (84). Six hours later (42 to 48 hours p.i.), both cultures were treated with the protein kinase G inhibitor compound 2 at a final concentration of 1 μM (85) to arrest parasite egress before exoneme/microneme secretion and therefore to enrich the cultures with mature schizonts of the same developmental stage and to reduce potential developmental delay effects.

After 5 hours of continued culture (47 to 53 hours p.i.), samples were taken for electron microscopy and immunofluorescence assay, and compound 2 was washed off to allow the continuation of parasite development to enable the determination of an invasion effect. Giemsa smears were prepared 0 to 6 hours, 36 to 42 hours, 42 to 48 hours, 47 to 53 hours, and 61 to 67 hours postinvasion. For the comparison to the endocytosis phenotype, rapalog was added already to the 0- to 6-hpi rings to inactivate PfVPS45 already at the beginning of the cycle.

### Indirect immunofluorescence analysis (IFA).

**T. gondii.** HFF monolayers grown on 24-well coverslips were infected with tachyzoites and grown for 24 hours. Immunofluorescence analysis was performed as previously described (86). Nuclei were stained with DAPI, and coverslips were mounted in Fluoromount G (Southern Biotech). Images were acquired with an LSM700 confocal scanning microscope (Zeiss).

**P. falciparum.** The IFA were carried out in solution essentially as described previously (87). The cells were fixed for 30 minutes at room temperature in 1× PBS containing 0.005% glutaraldehyde and 4% formaldehyde, washed 3 times in 1× PBS followed by permeabilization with 0.1% Triton X-100 for 10 minutes, and blocked in “blocking solution” (1× PBS containing 3% BSA, and 100 μg/ml ampicillin) for 1 hour at room temperature (RT). The samples were washed once in 1× PBS and incubated with rolling over night in blocking solution containing the respective primary antibody (rabbit anti-MSP1 diluted 1:1,000, mouse anti-GAPM2 1:500, mouse anti-RAP1 1:2,000, and mouse anti-AMA1 1:500) at 4°C. The next day, the cells were washed 3 times for 5 minutes in 1× PBS before addition of the secondary antibodies (Alexa Fluor 594 conjugated donkey anti-rabbit and Alexa Fluor 488 conjugated goat anti-mouse [Life Technologies]) diluted 1:2,000 in blocking solution. The samples were incubated for 1 hour with rolling at room temperature, and the cells were washed 3 times for 5 min in 1× PBS. Before imaging, the cells were seeded on a glass slide and covered with a coverslip.

### Western blotting.

Freshly lysed extracellular parasites were added to HFF monolayers. Parasites were harvested, washed once in PBS, and analyzed with the indicated antibodies as described previously (86).

### Plaque assays.

Freshly egressed parasites were inoculated on HFF monolayers. Parasites were grown for 7 days prior to fixation with 4% paraformaldehyde in PBS and stained with 0.1% crystal violet.

### Intracellular growth assay.

HFF monolayers were inoculated with freshly egressed parasites and incubated for 24 hours before fixation with 4% paraformaldehyde/0.005% glutaraldehyde in PBS. Cells were stained using a-GAP45 (1/5,000) to detect individual parasites. Results are shown as the mean and the standard deviation from 100 parasites counted in triplicate from three independent biological replicates.

### Invasion assays.

T. gondii tachyzoites were inoculated on HFF monolayers, centrifuged at 1,000 rpm, and allowed to invade for 30 min. HFF monolayers were subsequently fixed with 4% paraformaldehyde, and invasion was monitored by standard red/green invasion assays (60).

To calculate the rate of offspring per schizont P. falciparum stage, samples were taken immediately after the removal of compound 2 (47 to 53 hours p.i.) and 14 hours later (61 to 67 hours p.i.) to allow schizont rupture and merozoite invasion. The number of newly formed rings per schizont in comparison to the untreated control was calculated. Flow cytometry (FC) was used as previously described (84) to determine the parasitemia of the individual time points. For the measurement, 20 μl of the resuspended culture was taken and incubated for 20 min at room temperature with 80 μl of RPMI staining medium containing of 5 μg/ml Hoechst 33342 and 4.5 μg/ml dihydroethidium. After addition of 400 μl “stop-solution” (of 0.003% glutaraldehyde in cold RPMI), the parasitemia was determined with an LSRII flow cytometer by counting 100,000 events using the FACSDiva software. Statistical significance was determined by performing a two-tailed, unpaired *t* test.

### Rhoptry secretion phospho-STAT6 test.

Freshly egressed parasites, treated for 48 hours with rapamycin, were counted and resuspended in cold egress buffer at 20 × 10^5^ parasites/ml. Parasites were then used to infect HFF monolayers, and the coverslips were incubated for 20 minutes on ice. Egress buffer was changed by warm normal medium, and infected coverslips were incubated for 20 minutes in the water bath at 38°C. Parasites were fixed using cold methanol, and IFA was performed using anti-STAT-6 antibodies and DAPI.

### Evacuole secretion assay.

Rhoptry discharge was assessed using an evacuole detection assay (31). Freshly egressed parasites pretreated for 48 hours with or without rapamycin were first counted, washed in PBS, and resuspended in prechilled DMEM medium containing ±1 μM cytochalasin D followed by incubation on ice for 10 minutes. The parasites were then added to prechilled HFF-coated coverslips and incubated for 20 minutes on ice. The coverslips are then washed with cold PBS before adding complete DMEM medium containing ±1 μM cytochalasin D or DMSO, for controls, and further incubated for 20 minutes at 37°C in the water bath. Then, 4% paraformaldehyde was used to fix the parasites for 15 minutes. For this assay, ROP1 was used as a marker for rhoptry secretion (visualization of the evacuoles), while GAP45 was used to stain the pellicle of the parasites.

### Pulse invasion.

Freshly egressed parasites pretreated for 48 hours with or without rapamycin were resuspended in cold DMEM and kept on ice for 20 minutes after a 30-second centrifugation to settle them down on the host cell monolayer. Next, we incubated the parasites at 37°C for 3 minutes, and we fixed them with PFA for 15 minutes.

### Endocytosis assay.

The GFP-expressing plasmid was kindly provided by Mirco Schmolke. Endocytosis was assessed as previously described (20). Briefly, 1 × 10^6^ HEK293T cells (ATCC; CRL-3216) were seeded in 6-well plates, allowed to attach for 24 hours, transfected with GFP, and allowed to express the protein for another 24 hours before infection. Then, 5 × 10^6^ parasites were centrifuged at 1,000 rpm, allowed to invade for 10 minutes, and subsequently mechanically liberated by syringing. Parasites were fixed and analyzed. Treatment with morpholinurea-leucine-homophenylalanine-vinyl phenyl sulfone (LHVS) was performed for 2 hours on extracellular parasite (20 μM) and continued during infection of host cells (20 μM).

### Serial section transmission electron microscopy (ssTEM).

HFF cells infected with T. gondii parasites were grown in a monolayer on round (13 mm in diameter) glass coverslips, and Percoll-enriched pelleted Plasmodium falciparum parasites were fixed with 2.5% glutaraldehyde (Electron Microscopy Sciences) and 2% paraformaldehyde (Electron Microscopy Sciences) in 0.1 M sodium cacodylate buffer at pH 7.4 for 1 hour at room temperature. Cells were extensively washed with 0.1 M sodium cacodylate buffer, pH 7.4, and postfixed with 1% osmium tetroxide (Electron Microscopy Sciences) and 1.5% potassium ferrocyanide in 0.1 M sodium cacodylate buffer, pH 7.4, for 1 hour followed by 1% osmium tetroxide (Electron Microscopy Sciences) in 0.1 M sodium cacodylate buffer, pH 7.4, alone for 1 hour. Cells were then washed in double-distilled water twice for 5 minute each wash and stained with aqueous 1% uranyl acetate (Electron Microscopy Sciences) for 1 h. After a 5-minute wash in double-distilled water, cells were dehydrated in graded ethanol series (2 × 50%, 70%, 90%, 95% and 2 × absolute ethanol) for 10 minutes each wash. After dehydration, cells were infiltrated with a graded series of Durcupan resin (Electron Microscopy Sciences) diluted with ethanol at 1:2, 1:1, and 2:1 for 30 minutes each and twice with pure Durcupan for 30 minutes each. Cells were infiltrated with fresh Durcupan resin for an additional 2 hours. A coverslip with grown cells faced down was placed on a 1-mm-high silicone ring (used as spacer) filled with fresh resin, which was placed on a glass slide coated with mold-separating agent. This flat sandwich was then polymerized at 65°C for 24 hours in an oven. The glass coverslip was removed from the cured resin disk by immersing it alternately in hot (60°C) water and liquid nitrogen until the glass parted.

A laser microdissection microscope (Leica Microsystems) was used to outline the position of parasitophorous vacuoles on the exposed surface of the resin. The selected area was cut out from the disk using a single-edged razor blade and glued with superglue to a blank resin block. The cutting face was trimmed using a Leica Ultracut UCT microtome (Leica Microsystems) and a glass knife. Then, 70-nm ultrathin serial sections were cut with a diamond knife (DiATOME) and collected onto 2-mm single-slot copper grids (Electron Microscopy Sciences) coated with Formvar plastic support film. Sections were examined using a Tecnai 20 TEM (FEI) operating at an acceleration voltage of 80 kV and equipped with a side-mounted MegaView III CCD camera (Olympus Soft-Imaging Systems) controlled by iTEM acquisition software (Olympus Soft-Imaging Systems).
