# Supplementary figures and images for: The ZIP Code of Vesicle Trafficking in Apicomplexa: SEC1/Munc18 and SNARE Proteins
Source: mBio. 2020 Oct 20;11(5):e02092-20. doi: 10.1128/mBio.02092-20 (PMC7587439; doi:10.1128/mBio.02092-20)

Suppl Figure 1

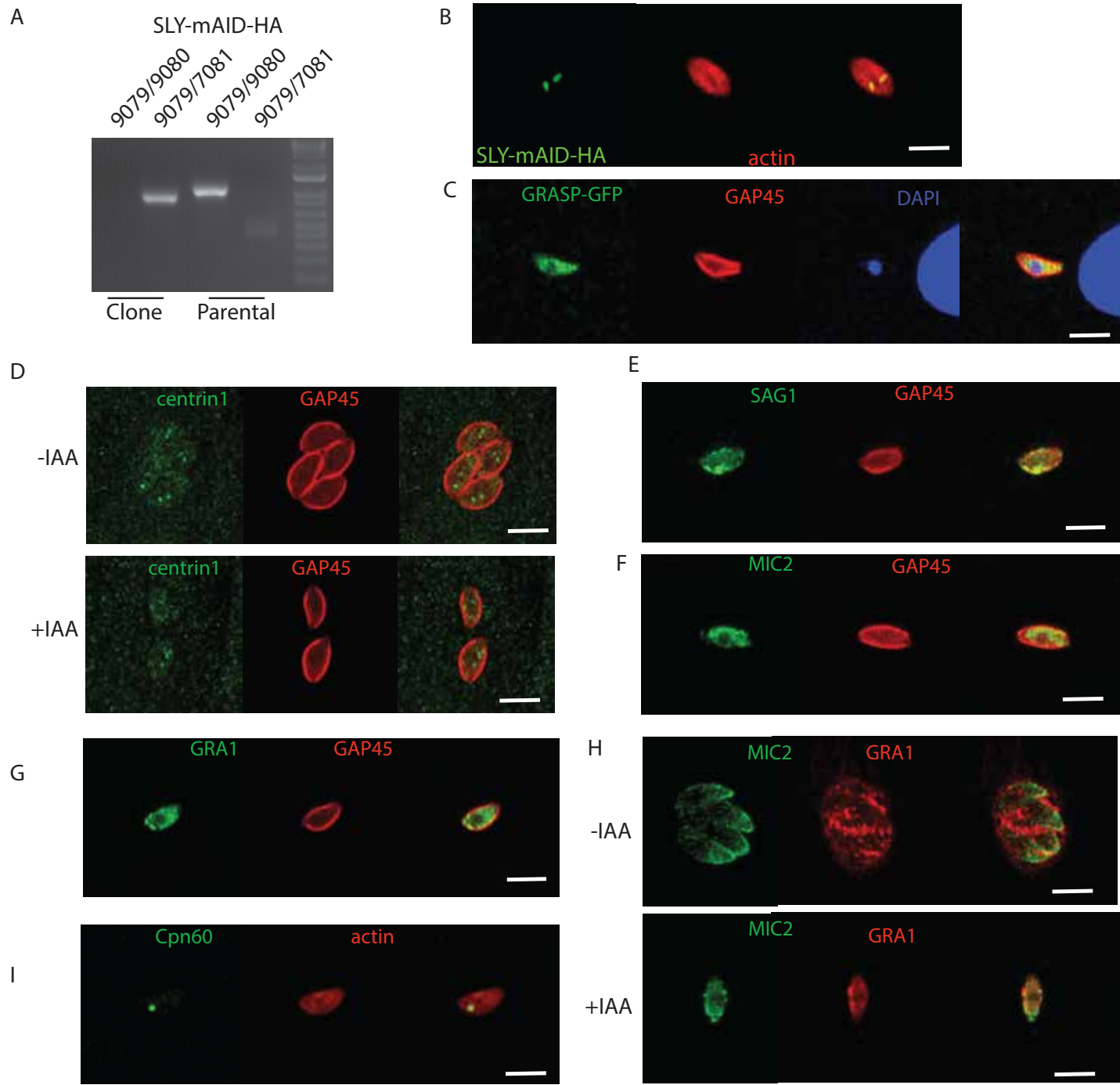

Supplement: FIG S1 [file mBio.02092-20-sf001.pdf]

**Suppl Figure 2**

**A**

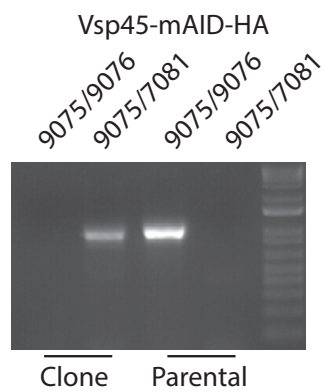

**B**

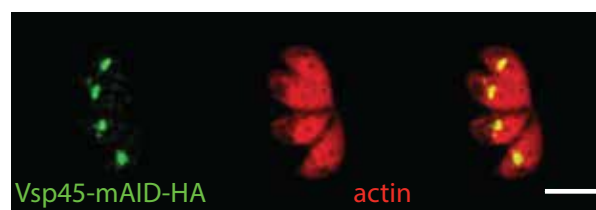

**C**

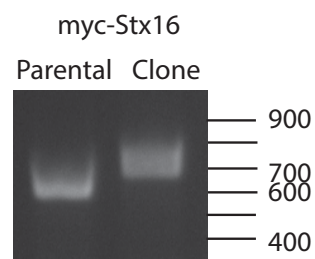

**D**

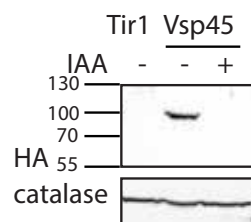

**E**

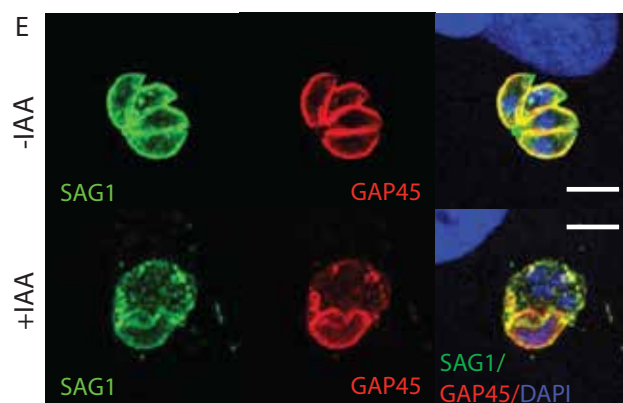

**F**

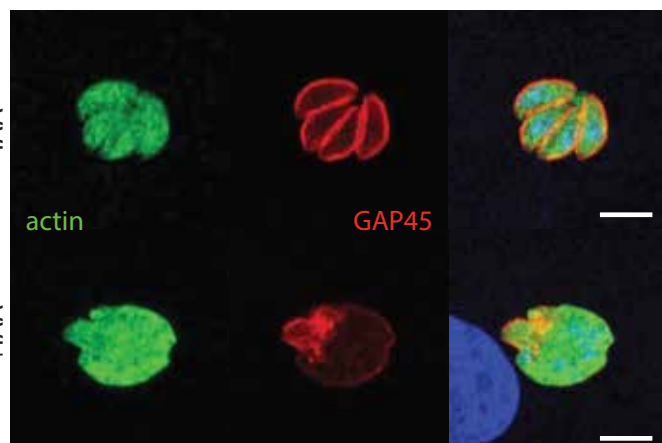

**G**

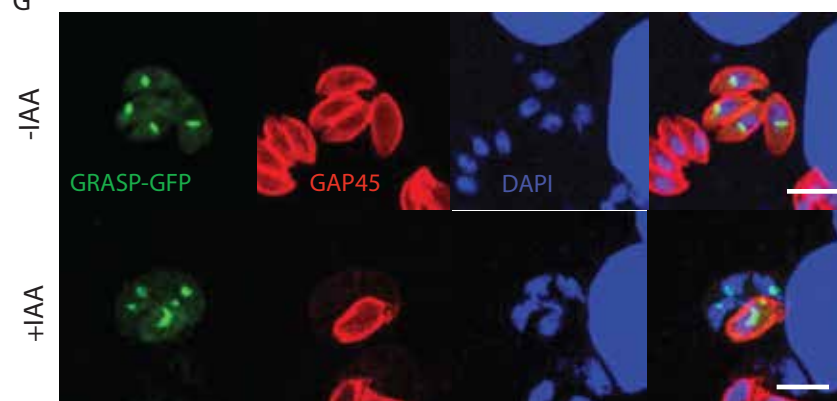

**H**

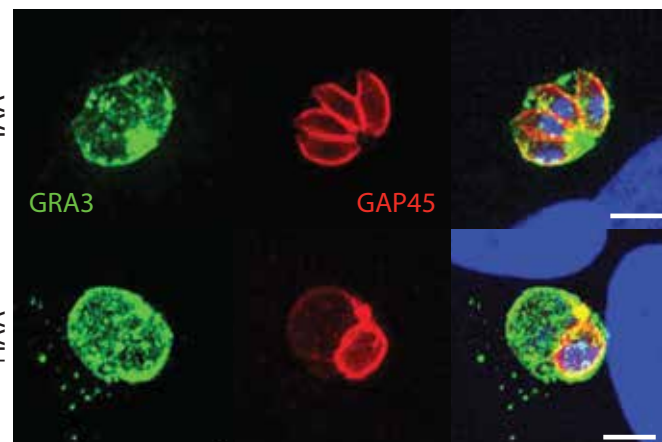

**I**

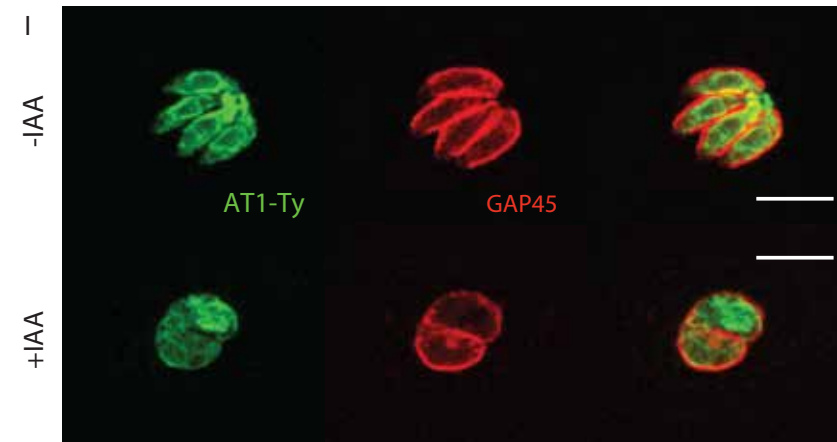

Supplement: FIG S2 [file mBio.02092-20-sf002.pdf]

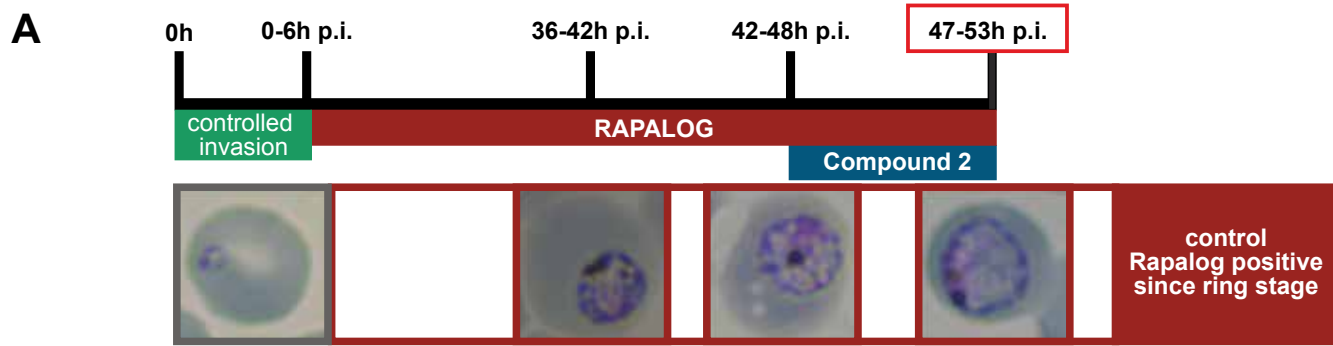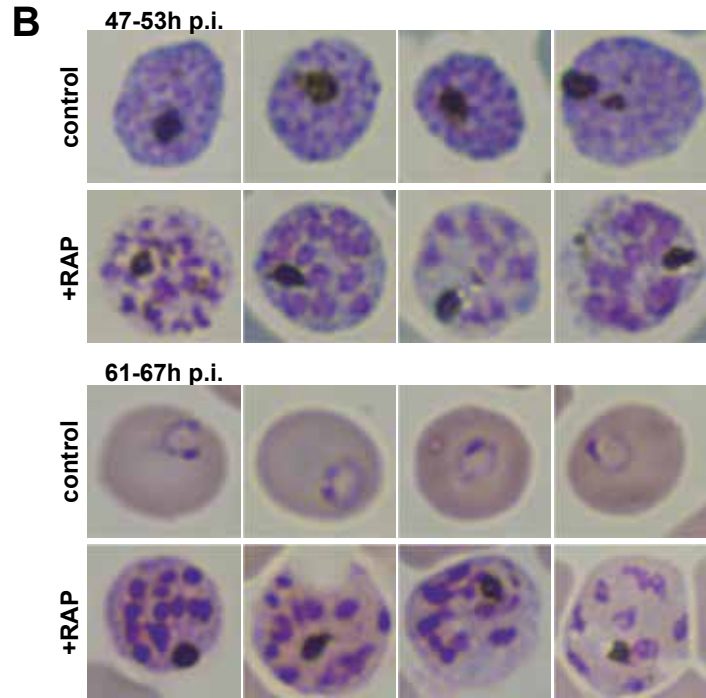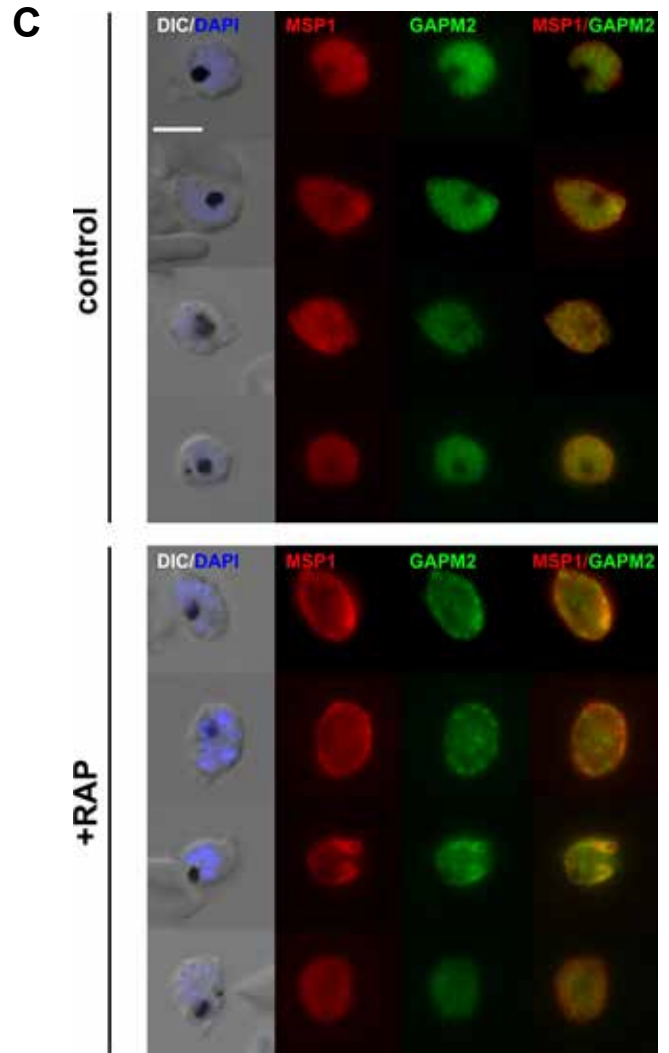

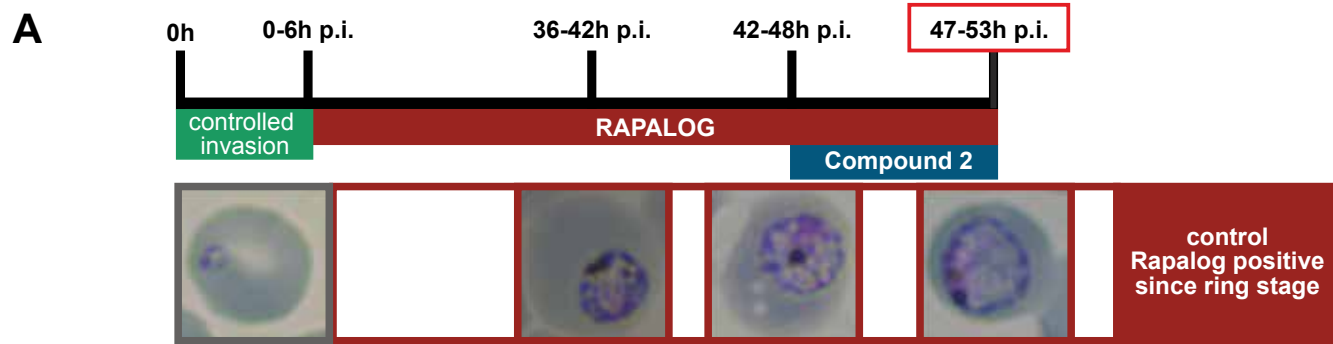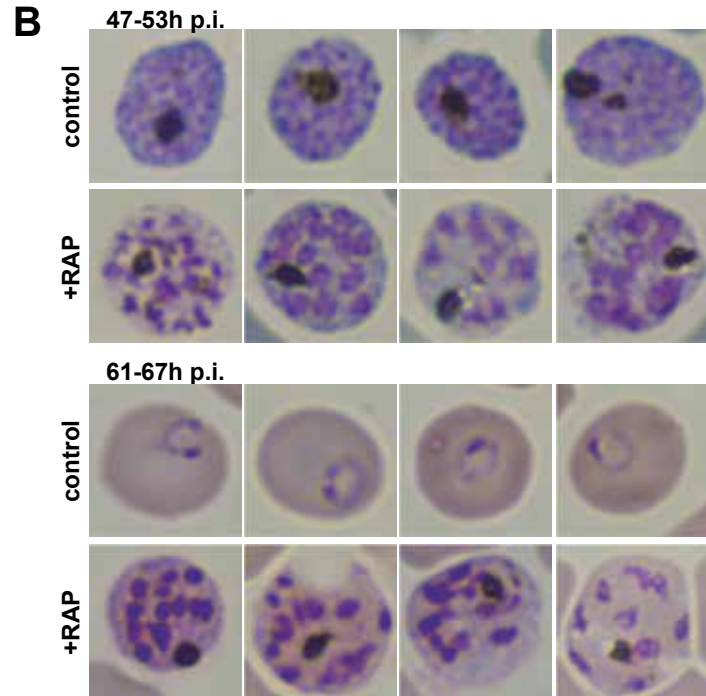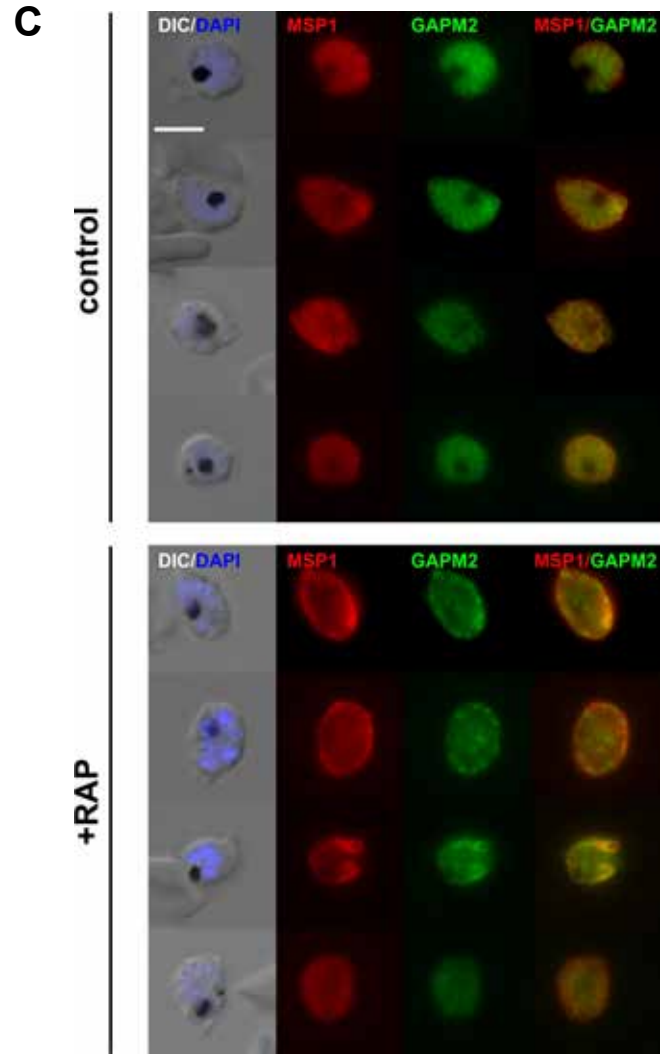

Supplement: FIG S3 [file mBio.02092-20-sf003.pdf]

A

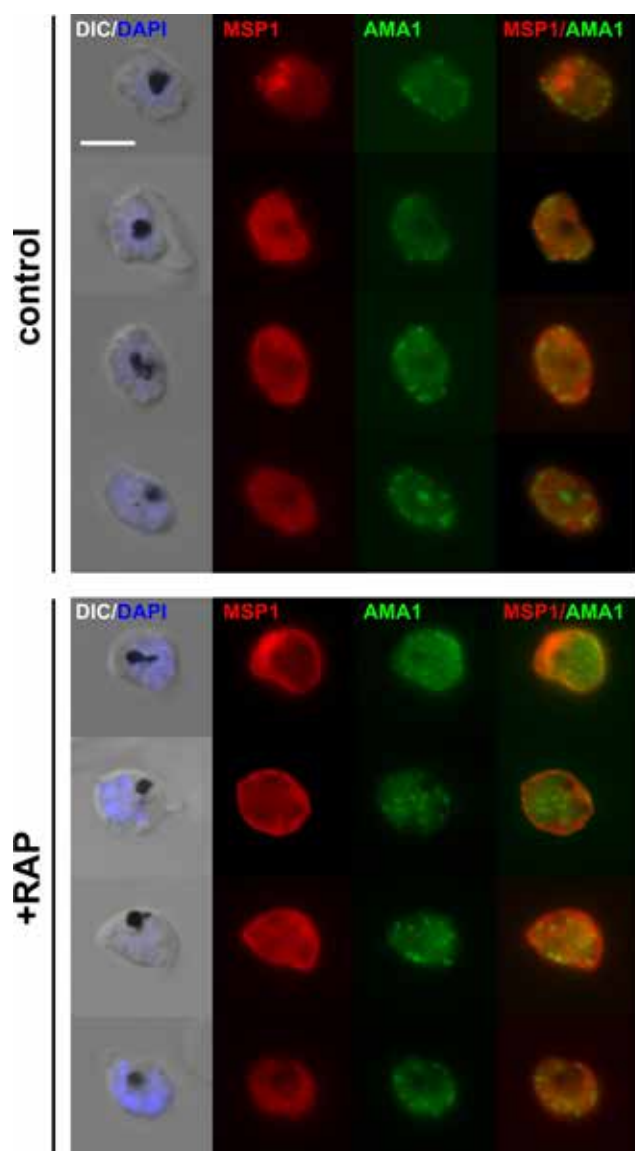

B

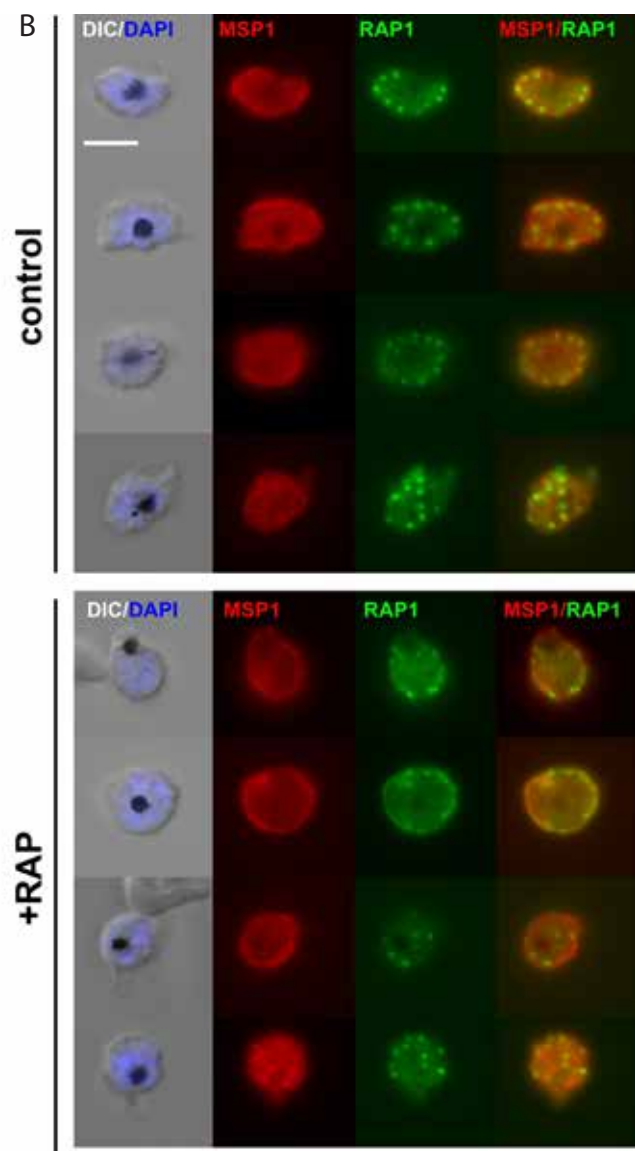

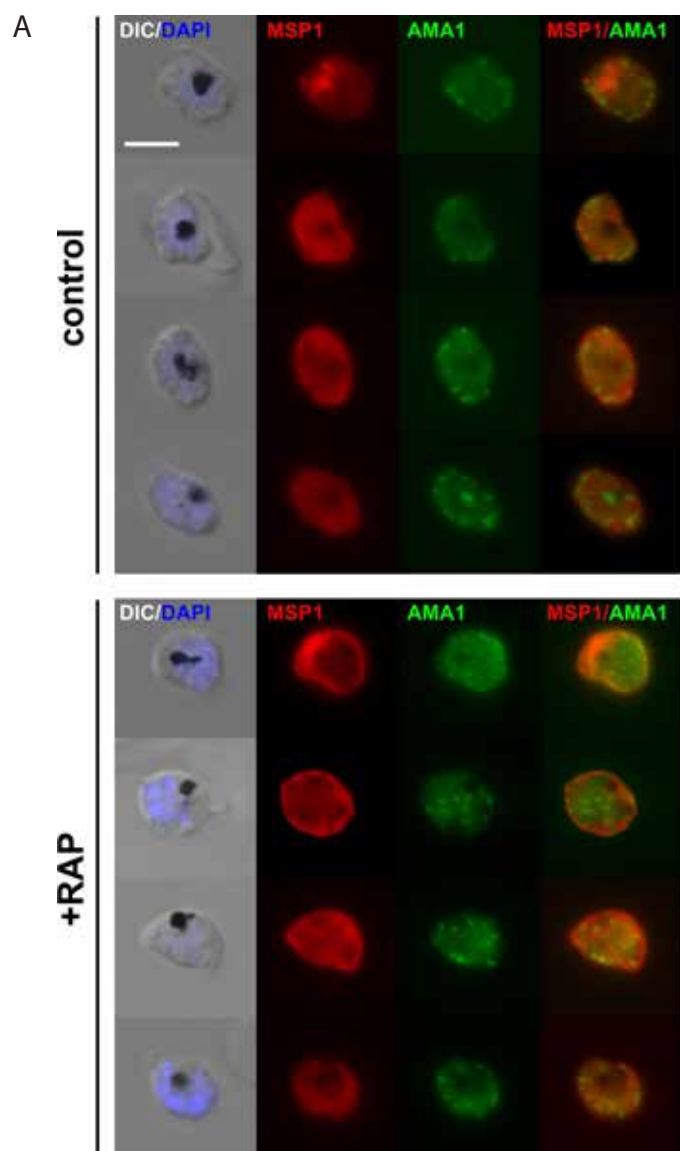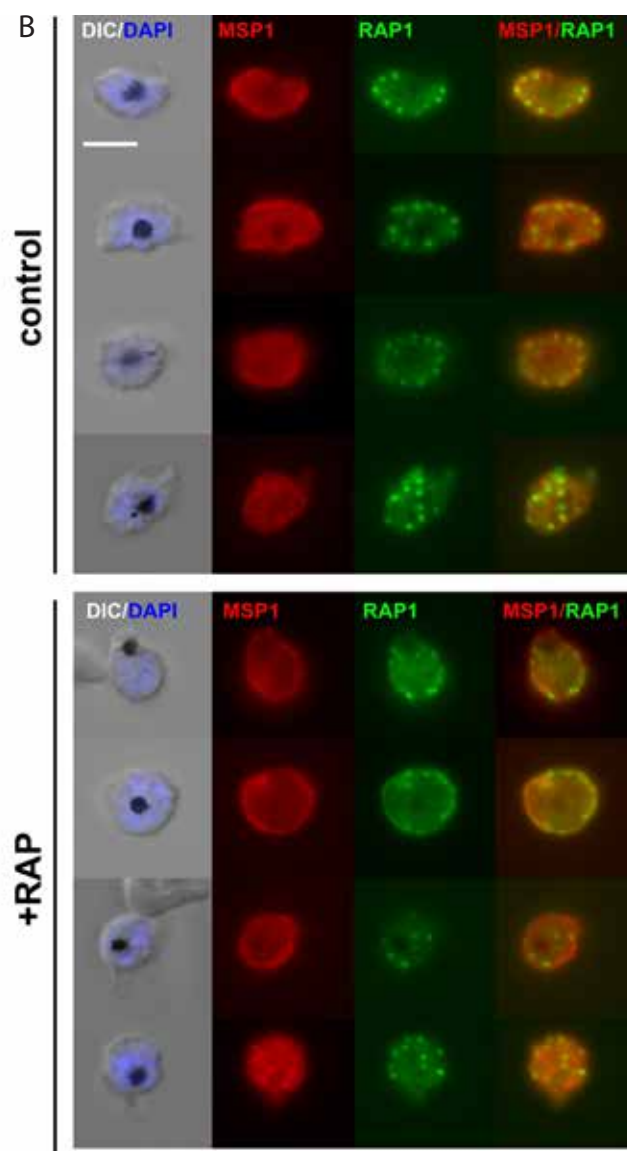

Supplement: FIG S4 [file mBio.02092-20-sf004.pdf]

Suppl Figure 5

A

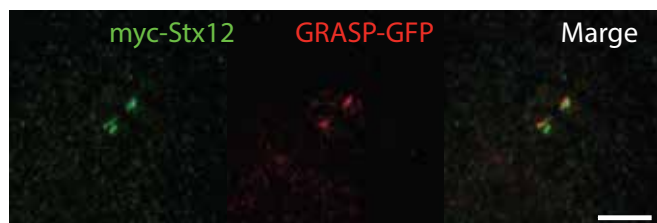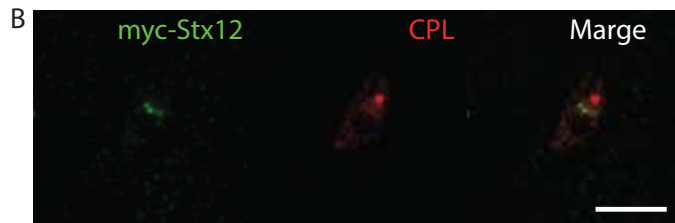

C

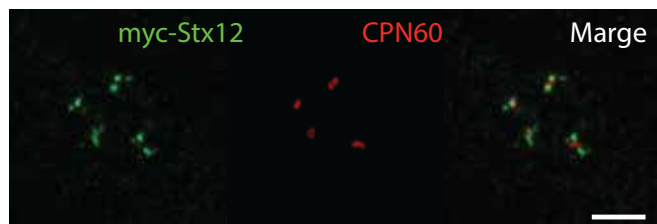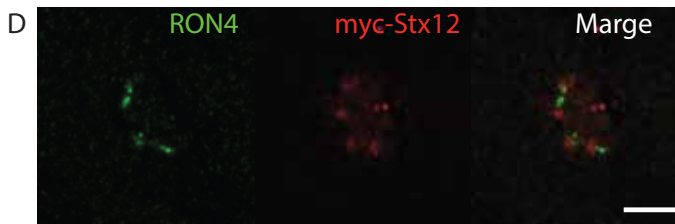

E

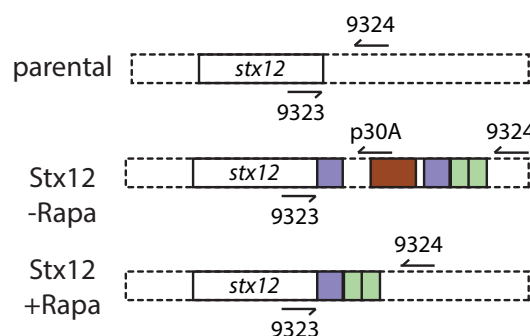

F

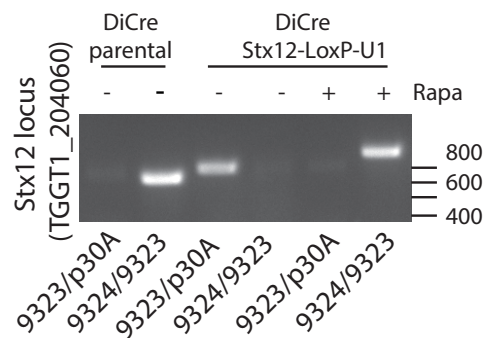

G

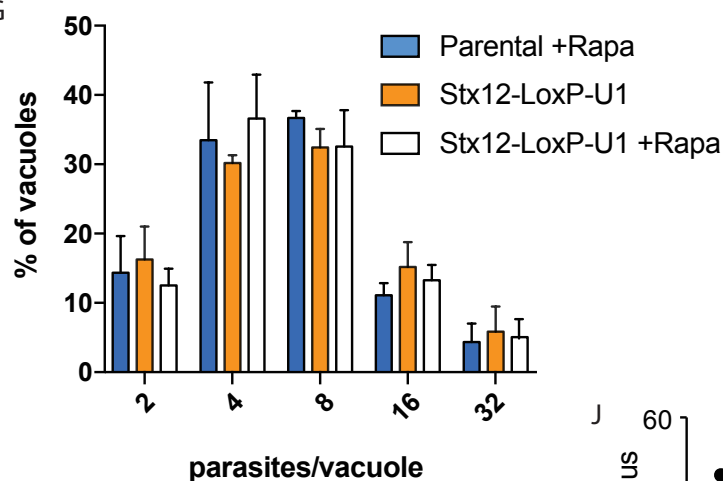

H

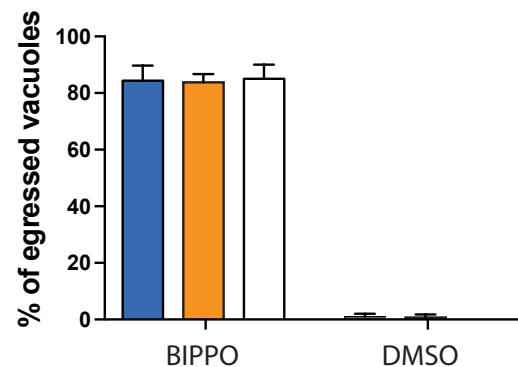

I

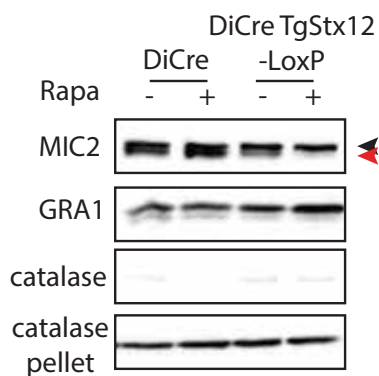

J

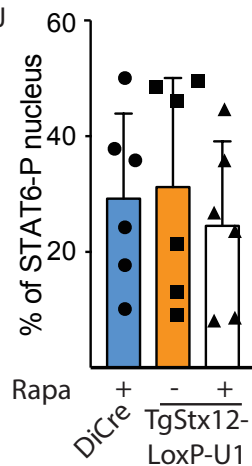

K

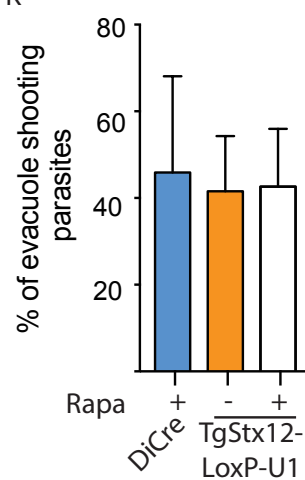

L

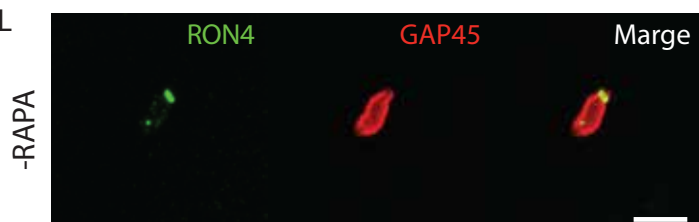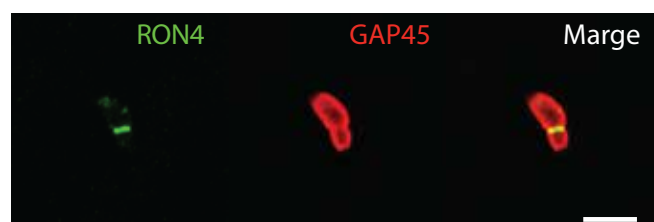

Supplement: FIG S5 [file mBio.02092-20-sf005.pdf]

**Suppl Figure 6**

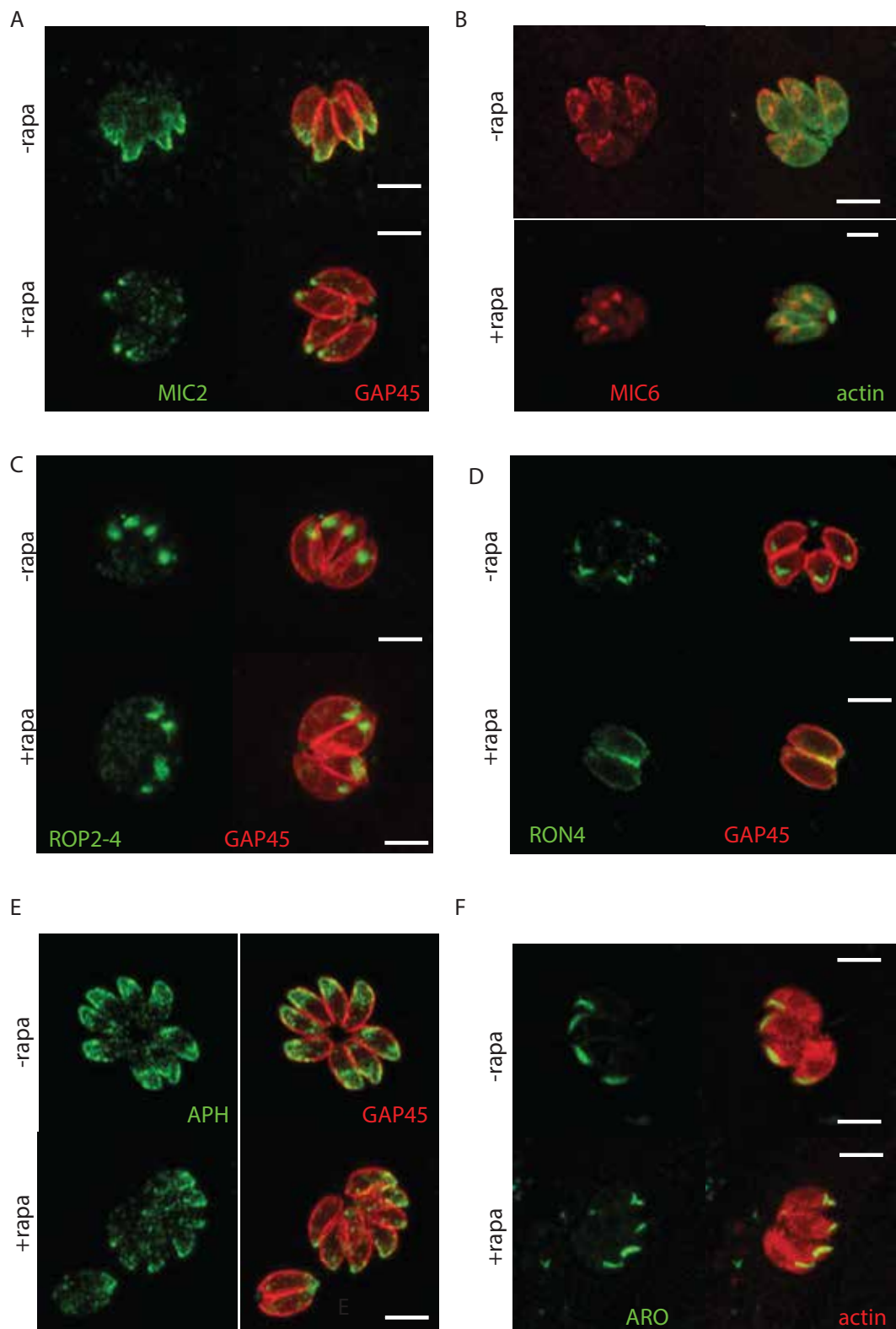

Supplement: FIG S6 [file mBio.02092-20-sf006.pdf]
