# Supplementary material for: The ZIP Code of Vesicle Trafficking in Apicomplexa: SEC1/Munc18 and SNARE Proteins
Source: mBio. 2020 Oct 20;11(5):e02092-20. doi: 10.1128/mBio.02092-20 (PMC7587439; doi:10.1128/mBio.02092-20)
Supplement: TABLE S1 [file mBio.02092-20-st001.docx]

**Supplementary Table 1.** Number of unique spectral counts detected for Vps45 interactors. PL: Protein length. TSP: Total spectral counts.

|  | **[Product Description]** | **[Gene ID]** | **[PL]** | **[TSC]** |
| --- | --- | --- | --- | --- |
| 1 | Ig gamma-1 | P01868 | 36 kDa | 388 |
| 2 | Immunoglobulin G-binding protein A | P02976 | 56 kDa | 362 |
| 3 | Trypsin | P00761 | 24 kDa | 33 |
| 4 | Keratin | P04264 | 66 kDa | 25 |
| 5 | Keratin | P13645 | 59 kDa | 22 |
| 6 | Keratin | P35527 | 62 kDa | 20 |
| 7 | Sec1 family protein | TGGT1_271060 | 73 kDa | 22 |
| 8 | Serum albumin | P02769 | 69 kDa | 10 |
| 9 | SNARE domain-containing protein | TGGT1_247930 | 36 kDa | 10 |
| 10 | Keratin | P15241 | 54 kDa | 9 |
| 11 | Keratin | P35908 | 65 kDa | 7 |
| 12 | rhoptry protein ROP5 | TGGT1_308090 | 61 kDa | 3 |
| 13 | Keratin | O43790 | 53 kDa | 5 |
| 14 | Keratin | O77727 | 49 kDa | 4 |
| 15 | Keratin | P25691 | 55 kDa | 6 |
| 16 | 2-oxo acid dehydrogenases acyltransferase | TGGT1_319920 | 70 kDa | 5 |
| 17 | Keratin | P02534 | 47 kDa | 2 |
| 18 | Keratin | P02539 | 13 kDa | 2 |
| 19 | Keratin-associated protein 6-1 | Q02958 | 8 kDa | 3 |
| 20 | putative SNAP protein | TGGT1_218760 | 36 kDa | 3 |
| 21 | hypothetical protein | TGGT1_241170 | 140 kDa | 3 |
| 22 | Keratin | P25690 | 46 kDa | 2 |
| 23 | ribosomal protein RPL3 | TGGT1_227360 | 44 kDa | 1 |
| 24 | rhoptry protein ROP7 | TGGT1_295110 | 63 kDa | 1 |
| 25 | Alpha-S1-casein | P02662 | 25 kDa | 2 |
| 26 | ribosomal protein RPS3A | TGGT1_232710 | 29 kDa | 2 |
| 27 | histone H2Ba | TGGT1_305160 | 13 kDa | 2 |
| 28 | actin | TGGT1_411760 | 32 kDa | 2 |
| 29 | arginyl-tRNA synthetase | TGGT1_270690 | 130 kDa | 2 |
| 30 | putative syntaxin 6 protein | TGGT1_300240 | 27 kDa | 1 |
| 31 | CorA family Mg2+ transporter protein | TGGT1_235402 | 128 kDa | 2 |
| 32 | ribosomal protein RPL18A | TGGT1_262670 | 21 kDa | 1 |
| 33 | ribosomal protein RPS4 | TGGT1_207440 | 36 kDa | 1 |
| 34 | Beta-casein | P02666 | 25 kDa | 1 |
| 35 | ROP18 | TGGT1_205250 | 62 kDa | 1 |
| 36 | ribosomal protein RPS6 | TGGT1_210690 | 29 kDa | 1 |
| 37 | ATP synthase beta subunit ATP-B | TGGT1_261950 | 60 kDa | 1 |
| 38 | ribosomal protein RPS14 | TGGT1_263700 | 16 kDa | 1 |
| 39 | putative elongation factor 1-alpha | TGGT1_286420A | 50 kDa | 1 |
| 40 | rhoptry neck protein RON5 | TGGT1_311470 | 187 kDa | 1 |
| 41 | rhoptry protein ROP8 | TGGT1_363030 | 64 kDa | 1 |
| 42 | histone H2A1 | TGGT1_261250 | 20 kDa | 1 |
| 43 | histone H4 | TGGT1_239260 | 11 kDa | 1 |
