# Supplementary material for: The ZIP Code of Vesicle Trafficking in Apicomplexa: SEC1/Munc18 and SNARE Proteins
Source: mBio. 2020 Oct 20;11(5):e02092-20. doi: 10.1128/mBio.02092-20 (PMC7587439; doi:10.1128/mBio.02092-20)
Supplement: TABLE S2 [file mBio.02092-20-st002.docx]

**Supplementary Table 2.** Primers used in this study.

| Primer | Sequence |
| --- | --- |
| **4883;** Universal reverse primer guide generation | AACTTGACATCCCCATTTAC |
| **7081;** Universal reverse primer mAID integration | GGGTCGAGCCCGAGCCCT |
| **8926;** Guide for SLY mAID tagging | GATGTCGAGGGCAGAGGAGTTGTTTTAGAGCTAGAAATAGC |
| **8927;** Forward primer for generating SLY mAID tagging | GGTGGGGTCCCCGACGCAGGAACCGCGTCTGCTAGCAAGGGCTCGGG |
| **8928;** Reverse primer for generating SLY mAID tagging | TCAGCTCGTCCCTGCTTTCTCTGTCGGTTCATACGACTCACTATAGGG |
| **8929;** Guide for Vsp45 mAID tagging | GCGCGAGATCTGCGCCGCAAAGTTTTAGAGCTAGAAATAGC |
| **8930;** Forward primer for generating Vsp45 mAID tagging | GCGTCGGAGGCCGACCTGCATGCGCGACCAGCTAGCAAGGGCTCGGG |
| **8931;** Reverse primer for generating Vsp45 mAID tagging | GGTTGTGCTGCATCCCGTGATCGACAGTCGATACGACTCACTATAGGG |
| **9075;** Forward primer check integration Vsp45-mAID | GCCGGCTGTCCACCGAACAG |
| **9076;** Reverse primer check integration Vsp45-mAID | GCTGAGTTAACCGGTGTTCG |
| **9079;** Forward primer check integration SLY-mAID | CGTTGGAGGGGGTTCCTTCG |
| **9080;** Reverse primer check integration SLY-mAID | CGTTCTCTTGCCAACGTCTC |
| **9303;** Guide for Stx6 cKD | GTCTGTTGCGCGTGAGACTTCGTTTTAGAGCTAGAAATAGC |
| **9304;** Forward primer for generating Stx6 cKD | TTACTTTTTCTTCTGCTCATCATCACGTAGCCTGCAGGATAACAGATAAC |
| **9305;** Reverse primer for generating Stx6 cKD | GTCTCAGTCTCTCCTCTCGAGTTCTCGAGCAGGAAACAGCTATGACCATG |
| **9306;** Guide for Stx12 cKD | GCTTCTGTTTCACCTCGCTGCGTTTTAGAGCTAGAAATAGC |
| **9307;** Forward primer for generating Stx12 cKD | CGGGGACTTCTGCCCCAGAGTCCGAACTAGCCTGCAGGATAACAGATAAC |
| **9308;** Reverse primer for generating Stx12 cKD | TGTCACTTCTTTCTCGCTTCTGTTTCACCCAGGAAACAGCTATGACCATG |
| **9423;** Forward primer check integration sxt12 cKD | CGTCCTTGTCTGCTGTCCTGCG |
| **9424;** Reverse primer check integration stx12 cKD | GCGCGAGGTCTGCATGTGC |
| **9420;** Forward primer check integration sxt6 cKD | GAGCGCCAATCGCCTGCACG |
| **9421;** Reverse primer check integration stx6 cKD | CAGTCGGCAGCGCCTGAAGG |
| **9424;** Guide for myc-Stx16 tagging | GAGACGCGCTCGACAGGATGGGTTTTAGAGCTAGAAATAGC |
| **9425;** Forward primer for generating myc-Stx16 tagging | AAAGAACTCGCAGACGCGCTCGACAGGATGGAACAAAAGCTTATTTCTGAAGAAG |
| **9426;** Reverse primer for generating myc-Stx16 tagging | GGTGATGTTCCTCGCTGCTAGTGTAGCCGCGAGATCCTCCTCGCTGATGAG |
| **9441;** Guide for AP2 tagging | GCTGGTCTCTGGGGTAGACTTGTTTTAGAGCTAGAAATAGC |
| **9442;** Forward primer for generating AP2 tagging | ACGCCGCAGAACTTCTACCGCATGACAACCGCTAGCAAGGGCTCGGG |
| **9443;** Reverse primer for generating AP2 tagging | CCAGCCGAGAGCGCTACAAATGTTAACAGCATACGACTCACTATAGG |
| **9499;** Forward primer check integration sxt16 tagging | CGGCTTCTCGCTCGTACCCAAG |
| **9500;** Reverse primer check integration stx16 tagging | GGGGAAGGCCATGACTGCTCTC |
| **9501;** Forward primer check integration AP2 tagging | GCTTGCGCGGATAACAGTGCG |
| **9502;** Reverse primer check integration AP2 tagging | CGGAGACCGCACGCGCG |
| **p30A;** Universal reverse primer check integration. | GTGACACCTGCAAGCCACAGCGG |
